# The Molecular Interplay Between p53-Mediated Ferroptosis and Non-Coding RNAs in Cancer

**DOI:** 10.3390/ijms26146588

**Published:** 2025-07-09

**Authors:** Carolina Punziano, Silvia Trombetti, Michela Grosso, Maria Lina Tornesello, Raffaella Faraonio

**Affiliations:** 1 Department of Molecular Medicine and Medical Biotechnology, University of Naples Federico II, 80131 Naples, Italy; carolina.punziano@unina.it (C.P.); silvia.trombetti@unina.it (S.T.); michela.grosso@unina.it (M.G.); 2 Molecular Biology and Viral Oncology Unit, Istituto Nazionale Tumori IRCCS Fondazione G. Pascale, 80131 Napoli, Italy

**Keywords:** p53, ferroptosis, lipid peroxidation, reactive oxygen species (ROS), glutathione, non-coding RNAs

## Abstract

Ferroptosis is a type of cell death executed by phospholipid peroxidation in an iron-dependent manner. Ferroptosis plays a central role in inhibiting tumor growth, enhancing the immune response, and is now considered a strategy to combat resistance to anticancer therapies. The oncosuppressor p53 is one of the major regulators of ferroptosis and can either promote or inhibit ferroptosis, depending on the context and/or extent of the damage. p53 governs the transcription of many genes that modulate cell susceptibility to ferroptosis, using this manner of death to fulfill its role as tumor suppressor. The diverse functions of p53 are related to non-coding RNAs (ncRNAs), especially microRNAs (miRNAs), and long non-coding RNAs (lncRNAs), since they can either regulate p53 or be regulated by p53. Therefore, an intricate metabolic network between ncRNAs and p53 ensures the correct response. In this review, we will discuss recent studies on the molecular interplay between p53-mediated ferroptosis and ncRNAs and how this contributes directly or indirectly to the outcome of ferroptosis.

## 1. Introduction

There is a large amount of data highlighting p53 as a critical factor in the regulation of ferroptosis, a type of cell death recently discovered and essentially driven by an excessive accumulation of phospholipid peroxides. Besides apoptosis, ferroptosis may represent another protective shield in p53-mediated tumor suppression. The intricate metabolic networks, comprising redox homeostasis, iron level regulation, mitochondrial activity, as well as the metabolism of lipids, and amino acids influence the occurrence of ferroptosis. In the context of ferroptosis, p53 displays a dual role as it can promote or even prevent ferroptosis, depending on the cellular and tissue context and on different stress factors as well as on the severity of stress and damage [1]. In fact, among the large repertoire of p53-dependent genes, there are several proteins/enzymes controlling the cellular levels of lipid peroxides, iron homeostasis, glutathione (GSH) amount, and the glutamine metabolism, as well as the prooxidant–antioxidant balance, all of which are important determinants in the mechanism of ferroptosis. Mounting evidence links p53 with non-coding RNAs (ncRNAs) [2], a class of endogenous transcripts that impact many physiological and/or pathological conditions. ncRNAs, including microRNAs (miRNAs), long non-coding RNAs (lncRNAs), and circular RNAs (circRNAs), are critical in the p53-mediated gene expression regulatory networks, since p53 activity/expression can be influenced by ncRNAs and in turn p53 as a transcription factor can mediate their expression [2].

This study presents a comprehensive update of p53’s role in ferroptosis with a particular emphasis on its molecular interplay with non-coding RNAs owing to ferroptosis surveillance.

## 2. Features and Mechanisms of Ferroptosis

Ferroptosis refers to a programmed cell death with distinctive morphological features different from apoptosis and other forms of death, which was first described in 2012 by Dixon et al. [3]. Although the chronological cascade is not well defined, ferroptosis is essentially executed by lethal lipid peroxides provoking irreversible membrane damage and consequent cell death. Pivotal conditions/factors causing ferroptosis include: (i) the accumulation of redox-reactive ferrous iron (Fe^2^) [3,4] that along with the increase in reactive oxygen species (ROS), in particular hydrogen peroxide (H_2_O_2_), can produce Fenton reactions [5,6,7]; (ii) the dysfunction of endogenous antioxidant systems, mainly glutathione (GSH) [3,8,9] and coenzyme Q (CoQ) [10], and (iii) the loss of enzymatic lipid repair, like glutathione peroxidase 4 (GPX4) [11].

(i) *Redox-reactive iron in ferroptosis.* Iron accumulation is an essential contributor to ferroptosis, as indicated by the term “ferroptosis” for this type of cell death. Iron pools that are directly involved in ferroptosis belong to (a) the cytosolic labile iron pool (LIP), which contains redox-active, non-coordinated Fe^2+^ that can be transported into mitochondria for the synthesis of iron-sulfur [Fe-S] clusters and heme [12,13]; (b) the iron pool used by iron-dependent enzymes such as lipoxygenases (LOXs) [14,15] and/or the iron-heme centers within the cytochrome P450 receiving electrons via NADPH cytochrome P450 oxidoreductase (POR) or NADH-cytochrome b5 reductase 1 (CYB5R1) [5,16]; and (c) the iron pool stored in the ferritin and released upon NCOA4-mediated ferritinophagy, the principal mechanism of iron increase during ferroptosis [4].

Ferrous iron is kinetically unstable and, in the presence of H_2_O_2,_ it can give rise to the non-enzymatic Fenton reactions that initiate and propagate lipid peroxidation (reviewed in [17,18]). Briefly, in this process, iron breaks down H_2_O_2_, a non-radical molecule, into the highly reactive intermediate hydroxyl radical (HO^•^) specie/s, that can react with the polyunsaturated fatty acids (PUFAs) of phospholipids (PL), initiating a series of radical-mediated chain reactions. By abstracting one allylic hydrogen, HO^•^ produces a carbon-centered lipid radical (L^•^) that rapidly reacts with O_2_ to generate a lipid peroxide radical (LOO^•^); this abstracts a hydrogen from an adjacent lipid chain producing lipid hydroperoxide (LOOH) and another L^•^ that continues the chain reaction. In the presence of iron, the O-O bond of LOOH is then decomposed in lipid-derived radicals which fragment into toxic malondialdehyde (MDA) and 4-hydroxynonenal (HNE). In line with this, Yan et al. demonstrated that the production of H_2_O_2_ through POR generated hydroxyl radicals (upon addition of iron), as revealed by specific electron spin resonance spectra, and assessed that HO^•^ radicals can abstract allylic hydrogens from PL-PUFAs that can initiate and propagate lipid peroxidation, with MDA production [5]. POR deficiency enhances resistance to drug-induced ferroptosis and decreases lipid peroxides without influencing the glutathione or GPX4 amount in most cancer cells [5,16]. In addition to POR, H_2_O_2_ production during ferroptosis can occur by specific enzymatic reactions performed by NOX enzymes [6,19], as well as during the mitochondrial election transport chain (ETC) (reviewed in [20]).

Non-heme iron is used by the enzymes arachidonate lipoxygenases (ALOXs) for the catalytic steps and redox cycle; ALOXs possess dioxygenation activity mainly towards arachidonic acid (AA, C20:4) and are considered pro-ferroptosis enzymes [14,15]; in fact, AA, along with linoleic acid (LA, C18:2) and adrenic acid (AdA, C22:4) is one of the major PL-PUFA targets involved in ferroptosis [21]. The human ALOX family comprises six isoforms (ALOXE3, ALOX5, ALOX12, ALOX12B, ALOX15 and ALOX15B) and all take part to ferroptosis induced by various drugs/conditions (reviewed in [22,23,24,25]). All the ALOX dioxygenases are linked to p53 either directly as target genes or indirectly [26,27] (see paragraph 5.1). Independent of the ALOX enzymes, another pro-ferroptosis cascade is represented by the acyl-coenzyme A synthetase long-chain family member 4 (ACSL4) and lysophosphatidylcholine acyltransferase 3 (LPCAT3) (ACSL4/LPCAT3) that are jointly required for activation and esterification of AA and AdA during the synthesis of phospholipids (reviewed in [28,29]). LPCAT3/ACSL4 knockout can protect lung adenocarcinoma cells from ferroptosis, while ectopic expression of LPCAT3 and ACSL4 has the opposite effect [30]. Doll et al. demonstrated that under GPX4 inactivation, only the absence of ACSL4, but no other ACSLs, could inhibit ferroptosis [31]. However, ACSL4 is not involved in p53-mediated ferroptosis upon ROS-induced stress [27]. Another source of PUFA-phospholipids in ferroptosis, includes the fatty acyl-CoA reductase 1 (FAR1), a peroxisomal enzyme producing fatty alcohols necessary for ether phospholipids (ePLs) biosynthesis (reviewed in [32,33]). The absence of FAR1 indeed enhances resistance to ferroptosis induced by erastin or RSL3 [34].

(ii) *Antioxidant systems and ferroptosis.* The primary antioxidant system protecting cells from ferroptosis is the solute carrier family 7 member 11 (SLC7A11)/GSH/GPX4 cascade [3,8,11,35,36]. It plays a central role in neutralizing lipid peroxides, hence preserving membrane integrity. The SLC7A11 transporter, a component of the xc_system, facilitates the import of cystine in exchange with glutamate [3,37]. The heterodimeric xc_system contains two subunits: SLC7A11 that confers specificity of the substrate and SLC3A2, less selective [38]. Once inside the cell, cystine is rapidly reduced to cysteine, the rate-limiting precursor for glutathione biosynthesis, a tripeptide composed of glutamate, cysteine, and glycine. GSH is linked to ferroptosis and the depletion of GSH is considered a key contributor in such a process, GSH being a necessary cofactor for the activity of GPX4 [3,39,40]. GSH biosynthesis involves two enzymes: the glutamate-cysteine ligase (GCL), containing the catalytic γ-glutamylcysteine synthetase (γ-GCS), and the glutamate-cysteine ligase modifier subunit (GCLM), and the glutathione synthase (GSS). Besides the GPXs, multiple antioxidant enzymes use glutathione as a cofactor, like GSH S-transferases and glutaredoxins, which act in iron cluster assembly and heme biosynthesis [41]. Moreover, GSH can react with free iron behaving as cytoplasmic buffering for ferrous iron [42] and GSH deficiency also increases the labile iron pool [43]. The coordinated activity of SLC7A11-mediated cystine uptake, GSH synthesis, and GPX4-dependent peroxidase function serves as a critical barrier against ferroptosis. Its disruption—via cystine deprivation, GSH depletion, or direct inhibition of GPX4—results in uncontrolled lipid peroxidation and ferroptosis induction [3,11]. Hence, the SLC7A11/GSH/GPX4 axis, by supervising redox homeostasis and PL-PUFA integrity, hampers ferroptosis. As stated before, cysteine availability, the levels of which are relatively low in cells [44,45,46]**,** is essential for GSH synthesis and mostly depends on SLC7A11 but also on de novo cysteine synthesis, via the transsulfuration (TSS) pathway [47,48]. The TSS pathway produces endogenous cysteine through the transfer of sulfur to serine and involves two enzymes: the cystathionine β-ynthase (CBS) and cystathionine γ-lyase (CGL/CSE) [47,48]. Another molecule that can counteract ferroptosis independently of GSH is the coenzyme Q10 (CoQ10) [10]. Traditionally recognized for its role as an electron carrier in the ETC, CoQ10 also functions as a potent lipophilic antioxidant, capable of preventing the accumulation of toxic lipid peroxides [10]. The link between CoQ10 and ferroptosis was clarified with the discovery of FIN56, a ferroptosis inducer that lowers GPX4 protein levels and depletes CoQ10 without affecting GSH levels [49]. A new link between CoQ10 and ferroptosis was provided by the identification of FSP1 (ferroptosis suppressor protein (1)), also known as AIFM2 (apoptosis-inducing factor mitochondria associated 2), a p53 target gene [10,50]. In fact, FSP1 reduces CoQ10 to ubiquinol (CoQH_2_), enabling the scavenging of LOO^•^ radicals at membranes, even in the absence of GPX4 [50]. This FSP1/CoQ10 system is also involved in the vitamin K redox cycle, producing VKH_2_, an additional antioxidant species [51]. In mitochondria, CoQH_2_ is generated by dihydroorotate dehydrogenase (DHODH) and glycerol-3-phosphate dehydrogenase 2 (GPD2) and suppresses mitochondrial lipid peroxidation in a manner complementary to mitochondrial GPX4 [52,53,54].

(iii) *Enzymatic lipid repair by GPX4.* GPX4 enzyme plays a pivotal role in membrane lipid repair. In 2014, a landmark study established that GPX4 overexpression protects cells from ferroptosis induced by multiple ferroptosis inducers (FINs), while its genetic ablation triggers a ferroptotic phenotype, characterized by lipid peroxide accumulation and organ dysfunction, such as acute renal failure [11,35]. As stated before, GPX4 enables the reduction of lipid hydroperoxides directly within cellular membranes, an activity unmatched by other glutathione peroxidase (GPX) isoforms or redox enzymes [36,55]. Moreover, unlike other GPXs, GPX4 exhibits a broad subcellular distribution, emphasizing its role in countering lipid peroxidation at key cellular sites [56]. The GPX4 mechanism of action relies on GSH as a cofactor [36]; hence, both GSH availability and cysteine metabolism (as a precursor of GSH) are critical for maintaining GPX4 activity, as elegantly demonstrated by early studies linking GSH depletion to ferroptosis sensitivity [3,39]. GPX4 knockout mice are embryonic lethal and GPX4-deficient cells accumulate high levels of lipid peroxides and rapidly undergo cell death under oxidative stress [57,58,59,60]. Interestingly, this lethality can be partially rescued by α-tocopherol, suggesting some functional overlap between lipid-soluble antioxidants [61]. Moreover, in cells lacking GPX4, PUFAs like AA and LA exacerbate lipid peroxidation and cell death, particularly when lipoxygenases (e.g., ALOX12/15) are active, implicating a GPX4-dependent regulatory axis over ferroptotic substrates [61,62]. Interestingly, direct GPX4 inhibitors, such as RSL3, ML162, ML210, FIN56, and FINO2, have demonstrated promising anticancer effects in many tumor types [11,63].

Data over the past years indicate that ferroptosis is involved in a number of pathophysiological processes [64,65,66] like neurodegeneration [67], blood disorders [68], kidney- and ischemia-reperfusion injuries [69,70]**,** senescence/aging [71]**,** as well as tumorigenesis [1]. In the cancer context, ferroptosis is mainly considered a tumor-suppressor mechanism and a novel therapeutic option to combat multiple forms of cancers, arresting tumor growth [66,72]. However, ferroptosis can exert an oncogenic function under specific conditions [73,74,75].

## 3. Non-Coding RNAs and Ferroptosis

Non-coding RNAs (ncRNAs) are a group of RNA molecules with limited protein-coding potential and relevant roles in the regulation of metabolism and other cellular processes that can be mainly achieved through the direct or indirect modulation of gene expression at transcriptional or post-transcriptional levels. In the last years, a growing body of evidence indicates that ncRNAs, especially miRNAs, lncRNAs, and circRNAs, play an important role in regulating tumorigenesis and the progression of various cancer types through epigenetic, transcriptional and translational regulation of ferroptosis-related gene expression, thus affecting the expression of a variety of ferroptosis-agonistic or -antagonistic effectors, including enzymes and other proteins involved in iron metabolism, glutathione metabolism, and lipid peroxidation as exhaustively reviewed elsewhere [76,77,78].

miRNAs are a group of small single-stranded ncRNAs of approximately 21–23 nucleotides that post-transcriptionally regulate gene expression by binding target mRNA sequences thus leading to translational inhibition or mRNA degradation and downmodulation of protein synthesis. In addition, more recently, it has been reported that miRNAs may also exert a role as translational activators to enhance protein expression [79,80,81,82,83].

LncRNAs are a class of heterogeneous ncRNAs, generally represented by transcripts of more than 200 nucleotides sharing many transcriptional and post-transcriptional features with mRNAs. LncRNAs mainly regulate cellular processes through the interaction with various other molecules, such as DNA, RNA, and proteins. Through these complex interactions, lncRNAs contribute to regulate gene expression by different mechanisms including epigenetic, transcriptional, translational, and post-translational processes (control of chromatin structure, methylation status, sequestration of miRNAs, assembly or disruption of protein complexes, and post-translational modifications) [84,85,86]. Some lncRNAs downregulate miRNAs by acting as a miRNA sponge and compete for microRNA binding to protein-coding transcripts. Furthermore, although being classified as ncRNA, mounting evidence even supports a protein-coding potential of lncRNAs that adds further complexity to the gene expression landscape. Notably, lncRNAs dysregulation is emerging as a cancer hallmark by affecting angiogenesis, cell metabolism and ferroptosis regulation, as outlined below [87,88].

CircRNAs are a class of single-stranded RNAs with covalently closed circular structures without a 5′ cap and a 3′ polyA tail that originate from exons via alternative mRNA splicing. CircRNAs have a high potential for gene regulation due to their higher stability than linear RNAs [89]. Several genes related to the iron, lipid and antioxidant metabolism are regulated by circRNAs that participate in ferroptosis modulation either as encoding proteins, modulators of gene expression or regulators of RNA splicing, although current research is mainly focused on the role of circRNAs as miRNA sponges [90]. This is the case of circKDM4C that sponges miR let-7b-5p to upregulate p53, thereby promoting p53-dependent ferroptosis pathways [91] and circRNA IL4R that inhibits miR-541-3p to enhance GPX4 expression, thus impairing ferroptosis in hepatocellular carcinoma [92]. In addition, other circRNAs, regulate ferroptosis by directly binding to proteins as in the case of the RNA-binding protein ALKBH5 (AlkB Homolog 5, RNA Demethylase), a negative regulator of ferritinophagy and ferroptosis, that is blocked by circ-cIARS [93].

In this review, we focus our attention on the interplay between ncRNAs and p53 in ferroptosis-mediated tumorigenesis and cancer progression with the aim of highlighting their agonistic and antagonistic effects on common regulatory pathways for the development of promising novel therapeutic strategies.

## 4. p53 in Cell-Cycle Arrest, Senescence and Apoptosis

The p53 protein is a fundamental transcription factor, widely known to have tumor suppressor activity, which is involved in the regulation of a broad range of cellular processes and essential in preventing aberrant proliferation and promoting cell death [94]. p53 represents a central node for creating a barrier against tumor initiation and development; hence, the functional inactivation of the p53 protein or mutations in the TP53 gene are crucial drivers of cancer progression [95]. Normally, p53 is expressed at low levels in the cells, due to constitutive ubiquitination and proteasomal degradation, mainly directed by the mouse double-minute 2 (MDM2), an E3 ubiquitin ligase [96,97]. Given that MDM2 is also a p53 target gene, an important feedback loop is generated between MDM2 and p53 [96,97]. Following stress, p53 becomes stabilized and transcriptionally active, able to recognize responsive elements located in the promoter regions of target genes [98,99,100]. In general, low/transient damage to DNA provokes cell-cycle arrest, allowing time to repair the damage, while intense/persistent stimuli activate the program of cell senescence; in the case of irreparable damage, cells program death pathways (apoptosis, autophagy, necrosis, as well as ferroptosis).

p53 drives cell-cycle arrest essentially by preventing the expression of the genes necessary for cell cycle progression [101]. At molecular level, this is achieved through an indirect mechanism, without binding p53 to the promoters of repressed genes. In fact, once activated, p53 directly induces the expression of p21/CDKN1A (cyclin-dependent kinase inhibitor 1A) [102] that inhibits the function of various cyclin/cyclin-dependent kinase (CDK) complexes coordinating cell proliferation. Among the substrates of CDKs, there are the tumor suppressor retinoblastoma (Rb) proteins, which control the activity of E2F transcription factors (E2F1-5) implicated in cell cycle progression. In general, Rb proteins in hypophosphorylated states selectively associate with and inactivate E2Fs [103]; when Rbs are phosphorylated by CDKs, their affinity towards E2Fs decreases, thereby leading to E2F-dependent activation of the genes needed for G1/S transition, DNA replication and cell division [102]. p53-dependent cell-cycle arrest is essentially reliant on p21 [104] that arrests cells at the G1-S [101] or G2-M phase [105] by fostering Rb-E2F repressive complexes. The p53 activation of p21 or MDM2 transcription is aided by CBP/p300 through the histone acetylation mechanism [106]. Of note, in cervical cancer, the human papillomavirus virus (HPV) E6 and E6associated protein (E6AP) potentiate p53 degradation [107] and SV40 large T antigen, HPV E7 or adenovirus E1A proteins bind to Rb and render E2F transcriptionally active with consequent proliferation [108].

p53 can drive senescence, a process known as an irreversible growth arrest protecting cells against oncogenic transformation but a causal factor in aging [109,110]. Briefly, DNA double-stranded breaks generated by stimuli of various natures (e.g., telomere shortening, oncogene activation, chemotherapeutic treatments) [111,112,113] induce a persistent DNA damage response (DDR) that through the kinases ATM (ataxia telangiectasia mutated) and ATR (ataxia telangiectasia and Rad3 related) [114] render p53 stabilized/activated and thereby able to initiate cascades converging on the two main senescence effectors: p53/p21 [109,115,116,117,118,119] and/or Rb/E2F/p16 [109,117,120,121,122]. Of note, p21 can create regulatory loops maintaining viability and impeding apoptosis [123] (and ferroptosis [124]). p53 can induce replicative senescence through the direct regulation of the plasminogen activator inhibitor-1 (PAI-1/SERPINE1) [125], arising from downregulation of PI(3)K/AKT–PKB signaling [126]. p53-mediated induction of p21 and PAI-1 is essential at the early stages of tumorigenesis for senescence-mediated growth arrest induced by the transforming growth factor-β (TGF-β) cascade [127,128]. PAI-1 transcription in vascular senescence seems to depend on the scaffold protein caveolin-1 [129,130,131] that prevents formation of the p53/MDM2 complex [132]. Numerous studies correlate senescence/aging with ferroptosis, given that age-related pathophysiological conditions like an iron increase in the organs can favor ferroptosis, albeit translating ferroptosis to the various clinical contexts in aging requires more investigation [71,133].

In non-transformed cells, p53 triggers apoptosis by direct transcriptional activation of genes encoding for the pro-apoptotic BH3-only proteins, especially PUMA and NOXA [134,135,136,137]. Given that mice carrying PUMA/NOXA double-knockout are more resistant to apoptosis than PUMA-null mice, these p53-dependent genes jointly contribute to apoptosis [138]. At molecular levels, PUMA and NOXA can bind and inhibit pro-survival/antiapoptotic proteins of the B cell lymphoma-2 (BCL-2) family comprising BCL2, BCLXL, and MCL1 [139]. This event promotes the release from BCLXL and MCL1, of BAX (Bcl-2-associated X protein) and BAK (Bcl-2 homologous antagonist killer), two cell death effectors, that activate the apoptosis pathway by regulating mitochondrial outer-membrane permeabilization and consequent activation of the caspase cascade [140]. In addition, p53 can migrate to mitochondria and directly interact with BCL-2 and BCL-XL antagonizing its function, thereby leading to the activation of effectors and ultimately apoptosis [141].

## 5. p53 and Ferroptosis

**Figure 1:**
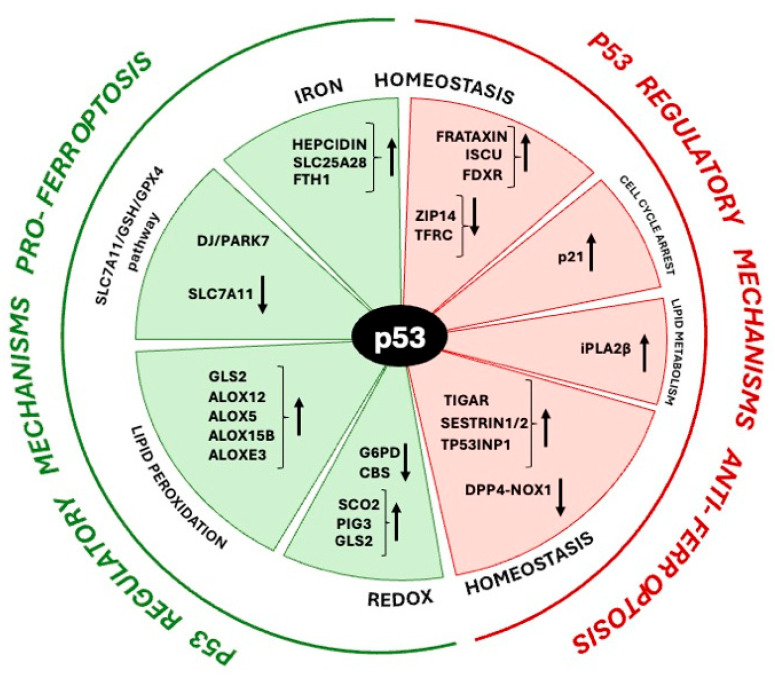
Schematic representation of the gene regulatory mechanisms mediated by p53 in ferroptosis. The effects of p53 (induction and repression) on the genes involved in ferroptosis are indicated with arrows. ALOX, arachidonate lipoxygenase; CBS, cystathionine β-synthase; DJ/PARK7, Parkinsonism-associated deglycase; DPP4, dipeptidyl peptidase-4; FDXR, ferredoxin reductase; FTH1, ferritin heavy chain; G6PD, glucose-6-phosphate dehydrogenase; GLS2, glutaminase 2; iPLA2β, Ca2+-independent phospholipase A2β; ISCU, iron-sulfur cluster assembly enzyme; NOX1, NADPH oxidase 1; p21/CDKN1A, cyclin-dependent kinase inhibitor 1A; SAT1, spermidine/spermine acetyltransferase 1; SCO2, synthesis of cytochrome C oxidase 2; SLC25A28, solute carrier family 25 member 28; SLC7A11, solute carrier family 7 member 11; TFRC, transferrin receptor; TIGAR, TP53 induced glycolysis regulatory phosphatase; TP53I3/PIG3, p53 inducible protein 3; TP53INP1, tumor protein p53-induced nuclear protein 1; ZIP14, iron-regulated transporter-like protein 14.

Emerging knowledge considers ferroptosis at the intersection of iron regulation, redox homeostasis and the metabolic pathways. Hence, considering the critical role of p53 in controlling such processes, it is not surprising that p53 regulates the susceptibility of cells to ferroptosis, using it to fulfill its role as a tumor suppressor along with cell-cycle arrest, senescence and apoptosis, and extensive research is ongoing to identify p53 target genes and downstream effectors in ferroptosis. The first evidence for a direct involvement of p53 in ferroptosis, dates back to 2015 from the work of Jiang et al. [142]. Actually, the evidence mainly points to a pro-ferroptosis role of p53 [142], even if it has been reported that p53 can also prevent ferroptosis, under certain conditions [124,143] and that some p53-dependent genes also seem to be under context-specific regulation [144]. The cascade initiated by p53 is multifunctional and as stated above, involves genes implicated in iron homeostasis, redox balance, and metabolic pathways. Figure 1 summarizes how p53, by supervising levels and/or functions of specific proteins linked to ferroptosis, has both pro-ferroptosis/pro-oxidant properties and pro-survival/antioxidant roles, as well as metabolic functions, and takes part in regulatory mechanisms exerting a dual role in ferroptosis, both as a promoter and inhibitor of ferroptosis, as we will discuss in this paragraph.

### 5.1. p53 in Pro-Ferroptosis Regulatory Mechanisms

*Iron homeostasis*. By regulating the expression of key genes implicated in iron metabolism both at systemic [145] and cellular levels [146,147,148,149,150], p53 is involved in the maintenance of iron balance. It has long been known that iron levels (as well as heme) can influence p53 activity: p53 expression can be decreased by iron excess [151], while p53 accumulates in conditions of iron depletion [152,153]. More recently, it has emerged that p53 itself can regulate iron levels by targeting iron regulators and that many iron regulators are important modulators of p53 activity. Firstly, p53 directly activates the expression of hepcidin/HAMP gene, encoding a liver peptide hormone that decreases circulating iron levels [145]; in fact, hepcidin blocks ferroportin1 (FPN1), the primary cell iron exporter [154], hence reducing serum iron but favoring intracellular iron accumulation that may induce ferroptosis; however, whether the p53-hepcidin-FPN1 axis plays a role in ferroptosis requires further investigation. The iron equilibrium could represent a part of defense against cancer operated by p53, as demonstrated in p53-deficient mouse-liver models of hepatocellular carcinoma displaying a status of iron deficiency associated with low hepcidin and a high transferrin receptor signature [155]. Iron homeostasis is important in living organisms [156]. In fact, iron is necessary for numerous processes like proliferation, DNA repair and energy, but it can also be potentially harmful, contributing to both tumor initiation and tumor growth (reviewed in [157,158]) as well as to ferroptosis-induced cell death, given that iron restriction can inhibit the growth of different types of tumors [159].

p53 can also regulate mitochondrial iron homeostasis via interaction with solute carrier family 25 member 28 (SLC25A28), also named mitoferrin 2, controlling iron trafficking to mitochondria [150]. A study in hepatic stellate cells revealed that bromodomain-containing protein 7 (BRD7) mediates the translocation of p53 to mitochondria, thereby facilitating its interaction with SLC25A28, that prolongs SLC25A28 half-live with consequent mitochondrial iron increase. This provoked hyperactivation of the electron transport chain and increased sensitivity to ferroptosis [150].

*Redox homeostasis.* p53 can modulate cellular redox status either by direct regulation of pro-oxidant and antioxidant gene expression or, indirectly, by modulating specific metabolic pathways [160,161]. Moreover, p53 itself is regulated by the cellular redox state [162,163] since the DNA binding of p53 is dependent on highly conserved domain owing to a zinc ion tetrahedrally coordinated with three cysteines and one histidine (reviewed in [164]). Several components of pro-oxidant cascades that can ultimately promote cell death by ferroptosis or apoptosis, are regulated by p53, either transcriptionally or post-transcriptionally. These comprise the TP53I3/PIG3 (p53 inducible protein 3), and SCO2 (synthesis of cytochrome C oxidase 2) as well as the metabolic genes GLS2 (glutaminase 2), G6PD (glucose-6-phosphate dehydrogenase), and SLC7A11 regulating NADPH and/or GSH levels.

TP53I3/PIG3 is a NADPH-dependent reductase that produces ROS [165]; this activity may be supported by its direct interaction with glutathione peroxidase 3 (GPX3) [166] or with association and inactivation of catalase, upon DNA damage [167]. Of note, catalase is a common interactor of p53 and PIG3 that by inactivating catalase, increases ROS levels [167]. However, a direct pro-ferroptosis effect of PIG3 has not been demonstrated yet. p53 directly activates transcription of the SCO2 gene [168]; it encodes a copper chaperone involved in the biogenesis of cytochrome c oxidase (COX), subunit II [169]. SCO2 fosters ROS generation and the dissociation of the apoptosis signal-regulating kinase 1 (ASK-1) from thioredoxin (Trx), thereby inducing an apoptosis cascade [170]. Since COX catalyzes the electron transfer from cytochrome c to molecular oxygen, SCO2 couples p53 with mitochondrial complexes/function [168]. However, recent data provide evidence that SCO2 proteins may be involved in oxidative stress defense and redox homeostasis rather than in ROS production [168]. Therefore, p53-dependent SCO2 function in ferroptosis needs to be further clarified. p53’s role in ferroptosis is also linked with GLS2 [171,172], a mitochondrial enzyme involved in glutaminolysis that converts glutamine into glutamate. p53 activates the transcription of GLS2 to modulate glutamine and GSH levels, decreasing ROS amounts [173]. However, under ferroptosis (GSH/GPX4 decrease), there is a shift in GLS2 activity: GLS2 catalyzes more production of α-ketoglutarate (using glutamate) that in turn activates the TCA cycle and ETC, thereby increasing lipid ROS; accordingly, xenograft tumors with GLS2 overexpression exhibit reduced tumor size and lower GLS2 in vivo correlates with hepatocellular carcinoma development [172]. Hence, the p53-GLS2 axis in this context acts as a tumor suppressor, differently from the isoenzyme GLS1 that is a cancer promoter [174]. Of note, a cancer-related mutation in p53 (P47S) fails to activate GLS2 (and SCO2) transcription and to evoke drug-induced ferroptosis, and mice carrying S47 are more prone to spontaneous cancers [175]. p53 interacts directly with glucose-6-phosphate dehydrogenase, a key regulator enzyme of the pentose phosphate pathway, and destroys the formation of the active dimer, hence lowering the production of NADPH [176] and potentially increasing sensitivity to ferroptosis. Cancer cells require high NADPH levels for both biosynthesis and ROS protection, hence control of NADPH amount is a part of the p53 tumor-suppressor function [176]. Finally, p53 is a negative transcriptional regulator of the SLC7A11 gene, lowering GSH synthesis and GPX4 activity [142,177]. This pro-oxidant pathway represents an important axis for a p53-mediated tumor-suppression function through ferroptosis [142,178]. Accordingly, in xenograft tumors, erastin-induced ferroptosis and tumor growth suppression provoked by p53 can be inhibited by SLC7A11 overexpression [142,178]. Of note, xc_system/SLC7A11 is frequently upregulated in cancer cells, enabling survival under stressful conditions and in ferroptosis [179]; inhibitors of the xc_system, like erastin and analogs (sulfasalazine, sorafenib), by interfering with x-CT function, provoke ferroptosis in most cancer cells [3,8] and thwart tumor growth [180] and in vivo data showed that increased SLC7A11 fosters primary tumor growth but suppresses tumor metastasis [179]. Furthermore, x-CT is a negative regulator of ALOXs, thereby lowering lipid peroxides [27,181]; therefore, it represents an attractive target for anticancer therapies (reviewed in [182]). Examples of recent studies focusing on p53-mediated SLC7A11 inhibition are reported hereafter. High expression of the acidic nuclear phosphoprotein 32 (ANP32E) was observed in esophageal cancer (EC) and ANP32E−/−cells showed increased ferroptosis upon erastin treatment, associated with increased p53-mediated SLC7A11 downregulation [183]. The absence of ANP32E renders cells more susceptible to paclitaxel treatments, and its combination with erastin lowered tumor growth both in vitro and in vivo [183]. Hongli et al. reported elevated levels of sex-determining region Y-related high-mobility group box 4 (SOX4) transcription factor in endometrial cancers. Given that SOX4 inhibits p53 by binding to p53 promoters [184], SOX4 knockdown renders cells more sensitive to ferroptosis via p53-dependent SLC7A11 inhibition; in fact, death was rescued by p53 depletion, suggesting a critical role of SOX4/p53/SLC7A11 cascade in ferroptosis [185]. In the context of cholangiocarcinoma, photodynamic therapy (PDT) results in an efficient approach to reduce tumor proliferation. It has been demonstrated that PDT upregulates p53 with subsequent suppression of SLC7A11, hence sensitizing cells to ferroptosis and the combination of PDT with ferroptosis inducers lowers tumor size in xenograft models [186]. Very recently, natural compounds comprising Tanshinone I, Ginsenoside Rh3, Curcumin, Resveratrol and Brazilin, have been tested for their capacity to induce ferroptosis via the p53-dependent pathway in different types of cancers, using in vitro and in vivo models. These compounds use as a common mechanism of drug-induced ferroptosis the downregulation of SLC7A11 dependent on p53 activation [187,188,189,190,191]. Resistance to ferroptosis can be achieved by interfering with the p53/SLC7A11 cascade. For example, in the context of mantle cell lymphoma (MCL), signal transducer and activator of transcription 5B (STAT5B) suppresses ferroptosis by promoting the transcription of DDB1 and CUL4-associated factor 13 (DCAF13) that in turn ubiquitinates p53, thereby increasing SLC7A11 expression and resistance to ferroptosis in MCL [192]. Gankyrin, a proteasomal chaperone, is a negative regulator of p53 by fostering MDM2-dependent p53 degradation, increasing malignancies and tumor progression [193]. Cells lacking gankyrin are more prone to erastin-induced ferroptosis, associated with increased p53 protein levels; gankyrin overexpression favors the MDM2-dependent ubiquitination of p53 with consequent upregulation of SLC7A11 and GPX4, thus facilitating cancer cell survival and impairing ferroptosis [193]. The transsulfuration pathway was linked to ferroptosis in a screening for genes causing resistance to erastin-induced ferroptosis that individuates the cysteinyl-tRNA synthetase (CARS) gene [47]. In lung cancer, the inactivation of p53 indirectly fosters CBS increase, hence inhibiting ferroptosis [194]. Accordingly, other studies demonstrated that CBS inactivation (by a selective inhibitor) enhances ferroptosis in hepatoma cells as well as in liver tumor xenograft mice models [48] and that erastin-induced ferroptosis can be counteracted by preserving homocysteine, a TSS-intermediate, through the DJ/PARK7 protein [195]. Given the established negative role of DJ/PARK7 on p53 binding activity (contributing to survival) [196], DJ-1 could interfere with p53-mediated ferroptosis, albeit no studies have been reported.

*Lipid peroxidation*. Peroxidation of PUFAs in membrane phospholipids is the causal factor of ferroptosis [197,198], but how peroxidation initiates the molecular cascades of such cell death is not clear [199,200]. As reported before, p53 controls ferroptosis sensitivity through the SLC7A11/GSH/GPX4 axis [39,55,142]. Qian et al. described a novel regulatory circuit involving interaction between mitochondrial p53 with hyperphosphorylated GPX4 [201]. Briefly, in sorafenib-induced ferroptosis, GPX4 was downregulated, mitochondria functionality compromised, and p53 accumulated into the nucleus, indicating that a retrograde signal of p53 can trigger ferroptosis. Moreover, p53 translocation from mitochondria to the nucleus was dependent on the dephosphorylation of GPX4 mediated by protein phosphatase PP2AB55β [201]. Accordingly, cells harboring constructs miming GPX4 dephosphorylation at serine 2 showed a decreased GPX4/p53 interaction and were more prone to ferroptosis; other data in vivo using xenograft tumor models, showed that overexpressing PP2AB55β-tumors had reduced growth and were more sensitive to sorafenib [201]. p53-mediated tumor suppression requires LOX activities participating in different types of death like apoptosis, autophagy, as well as ferroptosis [202]. It was demonstrated that under ROS-induced stress, p53-driven ferroptosis specifically required the ALOX12 enzyme, both in vitro and in vivo lymphoma models [27]. In this context, p53 by downregulating SLC7A11 expression indirectly fosters ALOX12 activation, given that SLC7A11 binds and sequesters ALOX12 [27]. A similar cascade involving p53/SLC7A11/ALOX15B activity was described in human bladder cancer, where ALOX15B and p53 expression were reduced. It was demonstrated that bladder cancer cells were sensitized to ferroptosis by p53 pharmacological activation or ALOX15B overexpression, whereas ALOX15B silencing had the opposite effect [181]. Another study in glioblastoma revealed that p53 indirectly promotes ALOXE3 activity through negative regulation of SLC7A11; ALOXE3 deficiency (caused by miR-18a) renders glioblastoma cells resistant to p53/SLC7A11-mediated ferroptosis, hence facilitating their growth in orthotopic models [203]. The ALOX15 isoform participates in oxidative stress-induced ferroptosis mediated by the p53/SAT1(spermidine/spermine acetyltransferase 1)/ALOX15 cascade [26]. In this case, p53 directly activates the transcription of the SAT1 gene, which specifically contributes to lipid peroxidation through ALOX15 activity, but not by ALOX12 or ALOX3. A link between the p53/SAT1/ALOX15 axis, ferroptosis failure, and resistance to cisplatin (DDP) has been reported in non-small cell lung cancer (NSCLC) [204]. DDP-resistant cells display an increased expression of HEAT repeat-containing protein 1 (HEATR1), a ribosome biogenesis factor; its silencing reactivates p53 with upregulation of SAT1 favoring ferroptosis through ALOX15 induction [204]. The ALOX5 gene in humans is a direct p53-responsive gene, induced in response to genotoxic stress; however, ALOX5 also acts in a negative feedback loop limiting p53 transcriptional activity through direct interaction [205]. Of note, cellular senescence due to oncogenic Ras or culture injury involves ALOX5, which activates p53 and its transcriptional target p21 [206].

Finally, ferroptosis mediated by ALOX enzymes was revealed in drug-induced ferroptosis such as erastin and RSL3, [25,207,208]. Erianin, an antipyretic/analgesic natural product induces ferroptosis in lung cancer (reviewed in [209]). It has been reported that Erianin, in a subset of carcinoma renal stem cells, promotes ferroptosis by enhancing m6A methylation on the mRNA of ALOX12 and p53, thus stabilizing their transcripts [210].

As mentioned before, p53 promotes the expression of the SAT1 gene during oxidative stress, DNA-damage injury or Nutlin-3 treatments (an MDM2 inhibitor), SAT1 being a direct p53 transcriptional target [26]. SAT1 is an enzyme implicated in polyamine catabolism and previous studies indicated that polyamine depletion triggers an intrinsic apoptotic pathway [211]. The first involvement of SAT1 in ferroptosis comes from the study of Qu et al. [26], who demonstrated that SAT1 was upregulated in p53-mediated ferroptosis and that elevated SAT1 correlates with high levels of ALOX15. The ferroptosis cascade mediated by SAT1 is independent of the SLC7A11/GPX4 axis and wild-type p53 can inhibit growth through a SAT1-mediated decrease in polyamines, Advanced studies also indicate that SAT1 is negatively regulated by the factor MAX’s Next Tango (MNT), a regulator of oncoprotein MYC, suggesting a complex network of SAT1 in ferroptosis [212].

### 5.2. p53 in Anti-Ferroptosis Regulatory Mechanisms

*Iron homeostasis*. Iron of LIP is mainly used by the mitochondria for iron-sulfur [Fe-S] clusters or heme. At cellular level, p53 directly upregulates the expression of iron-sulfur cluster assembly enzyme (ISCU) a scaffold component of iron-sulfur [Fe-S] cluster synthesis, that leads to increased cytosolic iron storage associated with decreased iron import [146]. At molecular level, high ISCU favors [Fe-S] cluster insertion into the iron regulatory protein 1 (IRP1), a condition that impedes the IRP1 from binding to the iron-responsive element (IRE) present in the 5′ UTR of ferritin heavy chain (FTH1) and in the 3′ UTR of transferrin receptor (TFRC) mRNAs, hence increasing translation of FTH1 and reducing TFRC levels [146]. Of note, a previous study by Zhang et al. reported that p53 provokes TFRC decrease, and ferritin increase at post-transcriptional level [213]. Since ferritin functions as a major iron storage protein and TFRC regulates iron entry into cells, the p53-ISCU pathway plays an important role in protection from iron overload and consequently from ferroptosis, albeit this latter point is missing in the literature. Zinc-regulated transporter and iron-regulated transporter-like Protein 14 (ZIP14 /SIc39a14) mediate the uptake of non-transferrin-bound iron [214]. It has been demonstrated that p53 directly interacts with and favors ZIP14 degradation; in fact its expression was increased upon loss of p53 and reduced under p53 overexpression [147]. Very recently, ZIP 14 was found upregulated and linked in vivo to hepatic ischemia and reperfusion injury, a pro-ferroptosis condition [215]. Therefore, it can be speculated that p53-ZIP14 interaction prevents ferroptosis. p53 enhances the transcription of the ferredoxin reductase (FDXR) gene, coding for a mitochondrial enzyme transferring electrons from NADPH to ferredoxins (FDX1/2), which participate in ISC biogenesis, essential for mitochondrial complex activities [216], steroidogenesis [217] and heme synthesis, thereby regulating iron levels in mitochondria and potentially preventing ferroptosis [218]. In fact, mouse models lacking the Fdxr gene showed embryonic lethality with the accumulation of iron; furthermore, Fdxr+/− mice presented an iron increase in the liver and were more prone to spontaneous tumors [148]. p53 is essential for the FDXR-mediated iron metabolism and, similar to other p53-target genes, the authors also found a mutual regulatory loop between p53 and FDRX in vivo and in vitro: the RNA binding protein IRP2 that is increased in FDXR deficiency acts as repressor of p53 mRNA translation with consequent defects in iron homeostasis [148]. Regarding ferroptosis (induced by RSL3 or erastin), both FDXR deficiency or overexpression suppressed this process, indicating that other mechanisms can influence this response [148]. In addition, p53 induces the expression of the frataxin gene [149], an iron-binding protein that mainly regulates [Fe-S] cluster biogenesis/assembly, essential for mitochondrial complex activities, thus preventing iron overload and ferroptosis [219]. There are studies indicating that frataxin may act as a tumor-suppressor protein, as demonstrated by using frataxin overexpression in colon cancer models (exhibiting growth inhibition) [220] or mice with disrupted hepatocyte frataxin expression (that develop liver tumors) [221]. However, frataxin could be increased in vivo in human glioblastoma tumors; by intersecting the p53 pathway, it can favor tumor progression/adaptation to hypoxic stress [222]. According to this, a recent study reported that mice overexpressing cardiac frataxin exhibited reduced myocardial ferroptosis during cardiac ischemia-reperfusion [223].

*Redox homeostasis.* Most of the p53-mediated antioxidant functions that counteract ferroptosis are mediated by the control of NADPH levels [163,224,225,226,227,228]. NADPH is mainly generated through the oxidative phase of the PPP pathway and by the malic enzymes, or the isocitrate dehydrogenases [229]. TIGAR (TP53-induced glycolysis regulatory phosphatase) is a metabolic p53 target gene encoding a fructose-bisphosphatase that hydrolyzes the fructose-2,6-bisphosphate, thereby directing glucose into the PPP with consequent NADPH production [138,140], inhibiting glycolysis and increasing PPP [230]. Accordingly, TIGAR increase has been associated with ferroptosis resistance in the development of colorectal cancer [231]. NADPH is an essential cofactor for the enzymes glutathione reductase (GSR) and thioredoxin reductase (TrxR), which regenerate GSH and Trx proteins, respectively, via a dithiol-disulfide mechanism (discussed in detail in other reports [232,233]). The thioredoxin/thioredoxin reductase (Trx/TrxR) system serves to recycle a variety of proteins [163], like peroxiredoxins, ribonucleotide reductase as well as transcription factors, including p53 [234]. Basal activity of p53 is positively influenced by the Trx/TrxR, either directly [235] or indirectly through Redox factor-1 (REF-1/APE, also called apurinic/apyrimidinic endodeoxyribonuclease 1) [236,237,238]. p53 is also linked to peroxiredoxin functions through the induction of the p53-activated gene 26 (PA26)/Sestrin1 [239] and the hypoxia-inducible gene 95 (Hi95)/Sestrin2 [240], which regenerate peroxiredoxins upon genotoxic and oxidative stress to lower ROS levels [241,242] eventually mitigating ferroptosis. However, a recent paper reported that sestrins boost the antioxidant Nrf2 pathway by favoring degradation of Keap1 via autophagy [243], hence counteracting the ferroptosis process [244]. The tumor protein p53-induced nuclear protein 1 (TP53INP1) is a p53 direct target gene [245] and different stressors can induce its expression [246,247]. It represents the major mediator of p53 antioxidant function [248]. TP53INP1 acts as a positive p53 cofactor enhancing its transcriptional activity through association with specific kinases [249,250]. However, a direct link of TP53INP1 with ferroptosis has not been established yet. Other genes regulated by p53 can directly participate in protection against ferroptosis. Tarangelo et al. demonstrated that stabilization of wild type p53 increases the expression of the *CDKN1A*/p21 gene which is causally implicated in the delay of ferroptosis onset induced by cystine deprivation in different types of human and mouse cancer cells [124]. The activation of the p53-p21 cascade in response to erastin fosters GSH conservation, leading to reduced toxic lipid ROS with consequent inhibition of ferroptosis [124]. Venkatesh et al., showed that cell lines with high p21 levels are more resistant to ferroptosis compared to those with lower p21 levels, independent of p53 [143]. The p21 protein is considered a limiting factor in cancer progression principally by inducing cellular senescence/aging [251]; however, p21 can also promote cancer survival, depending on the type of stressor as well as on tissue milieu [252]. For example, under serine starvation, transient induction of the p53–p21 axis also preserves GSH levels [253]. FSP1/AIFM2 is a direct p53 target gene, identified in human colon cancer cells during p53-dependent apoptosis [254]. FSP1 is a NAD(P)H-oxidoreductase [255] and plays a role in ferroptosis inhibition [10,50,51]; as reported before, it uses three GSH/GPX4-independent mechanisms: (i) the FSP1-CoQ10-NAD(P)H system reducing ubiquinone to ubiquinol that traps lipid peroxyl radicals [10]; (ii) the FSP1-VKH2-NAD(P)H pathway acting as reductase for vitamin K (VK), regenerating vitamin K hydroquinone (VKH2) that is a phospholipid radical-trapping antioxidant [51]; and (iii) the FSP1-ESCRT-III-dependent membrane repair system limiting lipid ROS [256]. Another mechanism by which p53 can prevent erastin-induced ferroptosis includes its interaction with dipeptidyl peptidase-4 (DPP4), in a transcription-independent manner [257]. DPP4, a mitochondria-encoded gene, induces ferroptosis through interaction with NADPH oxidase 1 (NOX1) [257]. p53, by favoring the localization of DPP4 toward a nuclear enzymatically inactive pool, leads to DPP4-NOX1 complex disruption and this decreases peroxidation/ferroptosis. Elimination of lipid peroxides represents an essential mechanism to counteract ferroptosis. A study reported that the Ca2+-independent phospholipase A2β (iPLA2β) is a direct p53-dependent gene. iPLA2β can remove oxidized lipid species embedded in membranes [258,259], hence preventing p53-driven ferroptosis due to tert-butyl hydroperoxide (TBH) exposure, independently of GPX4 [258]. In wild-type p53 cells, as well as in human melanoma A375 xenograft tumors, iPLA2β deficiency increased ROS-mediated ferroptosis. Molecular investigations revealed that iPLA2β protects from lipid peroxidation caused by the lipoxygenase ALOX12 [258]. Another investigation revealed that the iPLA2β protective function was due to its catalytic activity toward 15-HpETE-PEs generated by the ACSL4-LPCAT3-ALOX axis [259].

### 5.3. Regulation of Ferroptosis by Mutant p53

The p53 function is frequently disrupted in many human cancers by genetic alterations in the TP53 gene, which primarily include missense nucleotide substitutions [260]. The mutant p53 proteins are characterized by loss of function (LOF), dominant negative effect (DNE) on p53 tetramerization, or gain of function (GOF) [261,262,263]. In addition, such mutations are able to modify the activity of mutant p53 via epigenetic alterations such as the abrogation of phosphorylation, acetylation, methylation and ubiquitylation at specific sites [264].

Recent studies have shown that mutant p53 proteins are able to regulate several pathways including ferroptosis (Table 1). The initial observation was made in a mouse model carrying a lysine-to-arginine substitution at acetylation site K117 (p53K117R) in the p53 protein, which is able to mediate p53-driven cell-cycle arrest and senescence, but not apoptosis. Despite lack of apoptosis, the mouse did not develop spontaneous cancers [265]. In addition, the mutation of three acetylation sites in p53 (K117R, K161R, and K162R) resulted in the loss of its ability to transactivate most target genes and to induce cell-cycle arrest, senescence, and apoptosis, but the mice carrying triple-mutant p53 were not prone to developing spontaneous tumors [265]. Subsequent studies showed that triple mutant p53 (K117R, K161R, K162R) was able to reduce SLC7A11 expression and to induce ferroptosis upon exposure to t-butyl-hydroxide that fosters ROS production [142]. In addition, the ectopic expression of SLC7A11 was shown to promote tumor resistance to compounds inducing ferroptosis, confirming the significant role of SLC7A11 in the regulation of this pathway in cells harboring mutant p53 [266].

Conversely, mutations in four p53-acetylation sites (K98R/K117R/K161R/K162R), rendered the quadruple-mutant p53 unable to regulate the expression of genes involved in ferroptosis, including SLC7A11, or to control tumor growth [267].

An additional mouse model carrying the polymorphic variant p53P47S further supported the role of p53 variants in the regulation of ferroptosis [175]. The mutant p53P47S was able to activate cell-cycle arrest, senescence, and apoptosis in both human and mouse cells, but not ferroptosis, due to its inability to regulate GLS2 and SLC7A11. The mice carrying p53P47S were susceptible to developing tumors, mainly hepatocellular carcinomas and histiocytic sarcomas [175].

More recently, a humanized mouse model of triple-negative breast cancer carrying the p53R172H or p53R245W mutations, which can be switched on and off in tumor cells, has provided additional mechanisms on the regulation of ferroptosis [268]. In this model, deletion of the p53R172H or p53R245W mutations arrested tumor growth and significantly prolonged the survival of mice, demonstrating that these types of p53 mutants have anti-ferroptotic effects. The underlying mechanism was based on the ability of mutant p53 to enhance GPX4 activity, thus reducing lipid peroxidation and protecting cells from ferroptosis. In addition, the deletion of p53 mutations induced ferroptosis in breast adenocarcinomas via the expression of two peroxidase enzymes, MGST3 and PRDX6, which are dependent on the Nrf2 pathway [269].

Several types of human tumors harboring mutant p53 show decreased SLC7A11 levels and increased response to ferroptosis-inducing drugs [266]. For instance, the expression of SLC7A11 is reduced in esophageal and lung cancers through the interaction of mutant p53 with the transcription factor Nrf2, hence provoking an accumulation of ROS and the activation of ferroptosis [266]. Similar effects of mutant p53 on ferroptosis were detected in human colorectal cancer cells, where cells carrying mutant p53 showed increased sensitivity to erastin-induced ferroptosis compared to those expressing wild-type p53 [257]. Moreover, ectopic expression of the TP53 gene carrying the mutation R175H was shown to restore erastin sensitivity in both HCT116 and SW48 cells [257]. Therefore, it is essential to determine whether sensitizing cancer cells to ferroptosis inducers is a widespread phenomenon of different p53 mutants across various cancer types.

Several drugs have been developed with the ability to restore a wild-type-like structure of p53 mutants, the ability to induce the transcription of p53-dependent genes and to promote antitumor activity, inhibiting cell proliferation and tumor growth [260]. Among these, APR-246 is presently being investigated in phase I–III clinical trials in cancers that frequently show mutations in the TP53 gene, such as melanoma, esophageal cancer, high-grade serous ovarian cancer and myeloid malignancies [270,271,272].

In acute myeloid leukemia, APR-246 has been shown to induce cell death by promoting ferroptosis, independently of the TP53 mutational status [273]. Although APR-246 treatment enhances cystine uptake, GSH levels are decreased due to drug-induced depletion [266], leading to increased lipid peroxidation and ferroptosis induction.

In addition, APR-246 has been shown to synergize with factors inducing ferroptosis, such as chemical compounds or repression of SLC7A11 or GPX4 genes [273]. In conclusion, mutant p53 can regulate ferroptosis directly, by regulating the expression of genes involved in lipid peroxidation, iron metabolism, or antioxidant responses, or indirectly by enabling cancer cells to react to oxidative stress, metabolic changes, and other factors.

Ferroptosis has been shown to be a crucial process involved in the enhancement of tumor sensitivity to radiation therapy [274]. Indeed, radiation activates p53, which then suppresses SLC7A11 expression causing the repression of glutathione synthesis and an increase in lipid peroxidation, leading to ferroptosis. The p53 mutants that fail to suppress SLC7A11 allow cancer cells to become radioresistant. Importantly, ferroptosis inducers, which block SLC7A11, restore radiosensitivity in tumor organoids and patient-derived xenografts carrying p53 mutations or p53 loss [274].

Finally, in certain cancers, including head and neck squamous cell carcinoma, ferroptosis has a prognostic value based on the expression of ferroptosis-related genes and on p53-mutation status [275]. This model is able to predict the response to six cytotoxic drugs, and immune checkpoint inhibitor-related gene expression. However, further studies are needed on the role of p53 in ferroptosis regulation and its relationship with tumor treatment strategies, efficacy prediction, and prognosis assessment.

**Table 1:** Effects of p53 mutations on the regulation of ferroptosis in tumors.

p53 Mutation/s	Target/s	Effects on Ferroptosis	Cancer Type	Mechanism	Refs
K117R, K161R, K162R	SLC7A11	Pro-ferroptotic Agonist of p53	Spontaneous thymic lymphomas	Reduces SLC7A11 and induces ferroptosis under oxidative stress conditions.	[142,265]
K98R, K117R, K161R, K162R	SLC7A11	Anti-ferroptotic Antagonist of p53	Hepatocellular carcinoma	Prevents regulation of ferroptosis genes and tumor growth control.	[267]
P47S	GLS2, SLC7A11	Anti-ferroptotic Antagonist of p53	Hepatocellular carcinoma and histiocytic sarcoma	Unable to regulate ferroptosis targets; promotes tumor development in vivo.	[175]
R172H, R245W	GPX4, MGST3, PRDX6	Anti-ferroptotic Antagonist of p53	Mouse model of triple-negative breast	Stabilize GPX4 (↓ lipid peroxidation). Deletion induces ferroptosis via Nrf2-dependent enzymes.	[268,269]
R175H	DPP4	Pro-ferroptotic Agonist of p53	Human colorectal cancer cells	Restores erastin sensitivity in HCT116 and SW48 cells.	[257]
p53 missense mutations	SLC7A11	Pro-ferroptotic Agonist of p53	Esophageal and lung cancers	Reduces SLC7A11 expression by trapping Nrf2 and increases ferroptosis sensitivity.	[266]
R174Lfs*3, R248W, R248Q	SLC7A11, GPX4	Pro-ferroptotic Agonist of p53	Acute myeloid leukemia	Independently of TP53 mutation, APR-246 promotes ferroptosis.	[273]
p53 null or missense mutations	SLC7A11	Anti-ferroptotic Antagonist of p53	Solid tumors (NSCLC, osteosarcoma, breast and esophageal cancer)	Fails to repress SLC7A11 fostering radio resistance.	[274]

## 6. Interplay Between ncRNAs and p53 in Ferroptosis

**Figure 2:**
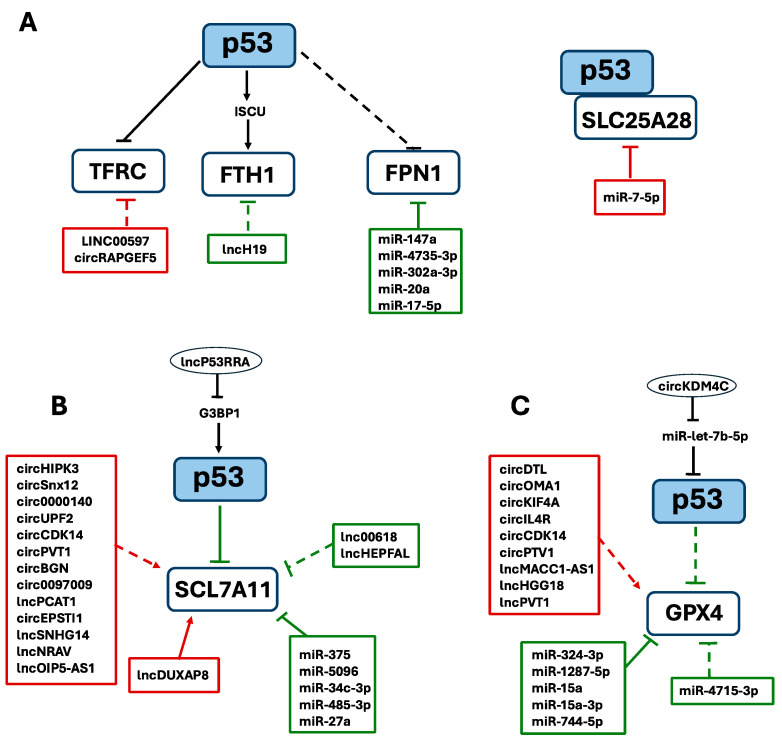
Molecular interplay between p53-mediated ferroptosis and non-coding RNAs in cancer. The ncRNAs influencing p53 or genes involved in ferroptosis are shown in red (suppressor role) and in green (activator role) boxes. Continuous lines indicate direct interactions; dashed lines indicate indirect interactions. (**A**) ncRNAs and p53 interplay in iron homeostasis; (**B**) ncRNAs and p53 interplay on SLC7A11; (**C**) ncRNAs and p53 interplay on GPX4.

Interplay can be considered a dynamic network of relationships between molecules that regulate each other or cooperate in a specific cellular process. In more detail, interplay between ncRNAs and p53 could include p53-mediated regulation of ncRNA expression; ncRNA-mediated regulation of p53 expression or function, like miRNAs that degrade p53 mRNA or inhibit its translation, or lncRNAs that stabilize or sequester p53; and ncRNAs that influence downstream targets of p53 or act in parallel modulatory pathways. In this review, a functional interplay has also been taken into account if an ncRNA acts on the same regulatory pathway as p53-for example, by regulating a target gene activated by p53 or modulating its functional activity-even if there is no direct interaction between p53 and the ncRNA. We thus focused our attention on ncRNAs able to exert mutual or coordinated influence in p53-dependent processes of ferroptosis-mediated tumorigenesis and cancer progression (Figure 2).

### 6.1. ncRNAs and p53 Interplay in Iron Homeostasis

As outlined above, p53 is involved in iron homeostasis by regulating the expression of several genes including hepcidin and ferroportin 1 (FPN1) [276]. Indeed, p53 enhances hepcidin levels and thus indirectly downmodulates FPN1 expression, hence increasing intracellular free iron accumulation and promoting ferroptosis [64]. A similar effect is exerted by a set of miRNAs including miR-124 in neuronal cells [277] as well as miR-147a, miR-4735-3p, miR-302a-3p, miR-20a and miR-17-5p in different types of cancers that downmodulate FPN1 by directly targeting its mRNA to block iron export and consequently promoting ferroptosis (Table 2) [278,279,280,281,282,283,284]. Conversely, miR-761 in a different context reduces hepcidin levels thus suppressing FPN1 degradation that in turn results in decreased iron deposition and reduced susceptibility to ferroptosis [285]. Another anti-ferroptotic miRNA is miR-7-5p that downregulates mitoferrin (SLC25A28) thus acting as an antagonist of p53 in the regulation of mitochondrial iron homeostasis in radioresistance [286] (Table 2). Moreover, miR-335 in Parkinson’s models, miR-224-5p in heart failure and miR-19b-3p in lung cancer directly downregulate FTH1 expression, thereby increasing ferroptosis [287,288,289]. In particular, curcumenol treatment by decreasing lncH19, provokes miR-19b-3p increase [289], hence it can be considered agonist of pro-ferroptosis p53. Furthermore, in different pathological contexts, miR-210-3p, miR-214 and miR-367-3p downmodulates TFRC expression thereby regulating ferroptosis execution [290,291,292,293]. Notably, cinobufotalin treatment increases LINC00597 expression which sponges miR-367-3p causing TFRC-mediated ferroptosis, thereby acting synergistically with p53 in lung cancer [292]. In addition, lncPVT1 by sponging miR-214 directly participates in p53-mediated iron homeostasis during brain ischemia/reperfusion. miR-214 binds the 3’ UTR of TFRC and since miR-214 could also bind 3’ UTRs of lncPVT1 and p53, a positive feedback loop between lncPVT1, miR-214 and p53 can be envisaged. lncPVT1 overexpression or miR-214 silencing significantly counteracted the effect of ferrostatin, a ferroptosis inhibitor [294], on ferroptosis in vitro [295]. More recently, two circRNAs were found to support p53-mediated ferroptosis: in heart failure circSnx12 was found to contribute to this complex network by acting as molecular sponge of miR-224-5p, whereas in endometrial cancer circRAPGEF5 downmodulates RBFOX2, an activator of TFRC, thus eventually promoting ferroptosis [90,288,296,297].

### 6.2. ncRNAs and p53 Interplay in Antioxidant Defense Systems

As already discussed, the vulnerability of cells to ferroptosis is mediated by lipid peroxidation induced by the imbalance of redox systems, caused by iron-dependent ROS accumulation and failure of antioxidant defenses, including glutathione levels and GPX4, which weaken the overall antioxidant cell capacity [17,299,300,301,302,303,304]. In this context, not surprisingly, antioxidant defenses are common targets of ferroptosis-regulating ncRNAs that may exert pleiotropic functions as up- or downregulators of key ferroptosis drivers, thus acting as tumor suppressors or oncogenes in several cancer types [305,306]. Given their relevance in ferroptosis-related processes, the expression levels of the two main effectors of the antioxidant defense systems, namely SLC7A11 and GPX4, appear to be regulated directly or indirectly by a large group of ncRNAs, as extensively reviewed in Table 3 and Table 4, respectively. Therefore, according to the significant role played by p53 in the regulation of the SLC7A11/ GSH/GPX4 axis, these ncRNAs may be classified as p53 agonists or antagonists based on their ability to promote or inhibit ferroptosis in cancer cells, thus demonstrating a strong and complex interplay of p53 and ncRNAs in the regulatory mechanisms of antioxidant defense systems [307,308].

### 6.3. NcRNAs and p53 Interplay in Lipid Metabolism

Many ncRNAs have been found to affect ferroptosis by regulating lipid metabolism. Indeed, lipids are important regulators of cell death, and the accumulation of lipid peroxides originating from PUFAs has a key role in promoting ferroptosis [21,197,344].

As reported before, the ALOX family represents the major class of enzymes for the oxygenation of arachidonic acid, one of the main PUFAs, thus eventually promoting lipid peroxidation and ferroptosis. Among the ALOX members, ALOX12 has been found to play a critical role in the pro-ferroptotic p53/SLC7A11/ALOX12 axis [27,345]. Therefore, by directly targeting and downmodulating ALOX12, miR-7-5p is able to inhibit lipid peroxidation and ferroptosis, thus acting as a p53 antagonist [346]. A similar effect is exerted by miR-522 on the expression levels of ALOX15, another member of the ALOX family, that participates to another mechanism triggered by p53, the p53/SLC7A11/ALOX15 cascade, as previously discussed [181,347]. Finally, another antagonist of p53 is miR-18a that directly binds to ALOXE3 mRNA and represses its expression to inhibit ferroptosis [203,300]. An important role in the PUFA metabolism is played by ACSL4, as mentioned before, a classic driver of ferroptosis, targeted by several ncRNAs. However, ACSL4 has not been reported as being required for p53-mediated ferroptosis [348,349,350].

## 7. Conclusions

This review covers the current state-of-the-art of the interplay between p53 and ncRNAs in the regulation of ferroptosis in different cancer cells and offers a glimpse into the unexplored landscape of opportunities, challenges and potential clinical applications raised by these complex interactions.

In recent years, ncRNAs have emerged as fine-tune regulators of gene expression, with growing evidence indicating their dysregulation involvement in cancer development and progression. Indeed, tumor-suppressive ncRNAs are often downregulated in cancers, leading to the de-repression of oncogenes [351]**,** whereas, conversely, oncogenic ncRNAs can promote tumorigenesis by silencing tumor-suppressor genes [352]. Consequently, ncRNA-based therapeutics in cancer treatment are increasingly being explored due to their ability to target multiple genes and pathways simultaneously.

Furthermore, the role of ncRNAs in drug resistance has raised great interest in the identification of new targets and modalities for cancer treatment. However, although the compatibility of ncRNA-based treatments with nanotechnology-targeted delivery systems further supports their translational potential, significant challenges still remain to be addressed in developing effective ncRNA-based therapeutics [353].

Therefore, this review provides a comprehensive overview of the recent advancements in the interplay between p53 and ncRNAs for a better understanding of the molecular mechanisms and regulatory pathways of ferroptosis in cancer, thus shedding novel light on current challenges and the future directions of ferroptosis in the treatment of cancer.

In summary, although many issues remain to be addressed in this rapidly evolving field, great attention should be paid in future research to gain more comprehensive knowledge on p53 and its agonists and antagonists in ferroptosis to reap more fruitful avenues for both research and clinical applications.

## Figures and Tables

**Table 2:** ncRNA and regulation of iron homeostasis: impact on ferroptosis and p53 axis in tumors.

ncRNAs	Target	Cancer Type and Tumor Significance	Mechanism of Action on Iron Homeostasis	Refs
circRAPGEF5	RBFOX2	Endometrial cancer Tumor suppressor	It downmodulates RBFOX2 and activates TFRC.	[298]
lncH19	miR-19b-3p	Lung cancer Tumor suppressor	It sponges miR-19b-3p that directly targets FTH1.	[289]
LINC00597	miR-367-3p	Lung cancer Oncogene	It sponges miR-367-3p that directly targets TFRC.	[292]
miR-147a, miR-4735-3p, miR-302a-3p miR-20a, miR-17-5p	FPN1	Glioblastoma, clear cell renal carcinoma, non-small cell lung cancer, lung cancer and multiple myeloma Tumor suppressor	They directly downregulate FPN1 expression.	[278,279,280,281,282]
miR-7-5p	Mitoferrin (SLC25A28)	Radioresistant cells from different cancers Oncogene	It downmodulates mitoferrin (SLC25A28).	[286]

**Table 3:** ncRNAs in the regulation of ferroptosis and of the p53-SLC7A11 axis in tumors.

ncRNA	Target	Cancer Type and Tumor Significance	Mechanism of Action on SLC7A11	Refs
circHIPK3	miR-508-3p	Gastric cancer Oncogene	It sequesters miR-508-3p that directly targets Bcl-2, finally resulting in SLC7A11 increase.	[309]
circSnx12	miR-194-5p	Ovarian cancer Oncogene	It sponges miR-194-5p that directly inhibits SLC7A11 expression.	[310]
circ0000140	miR-527	Oral squamous cell carcinoma Oncogene	It reduces miR-527, preventing it from exercising negative regulation of SLC7A11.	[311]
circUPF2	IGF2BP2	Hepatocellular carcinoma Oncogene	It forms a ternary complex with IGF2BP2 stabilizing SLC7A11 mRNA.	[312]
circCDK14	miR-3938	Glioma Oncogene	It sponges miR-3938, which directly suppresses PDGFRA expression associated to subsequent SLC7A11 increase.	[313]
circPVT1	miR-30a-5p	Esophageal squamous cell carcinoma Oncogene	It sponges miR-30a-5p which negatively regulates the FZD3 receptor, indirectly increasing SLC7A11 expression.	[314]
circBGN	OTUB1	Breast cancer Oncogene	It acts as a molecular scaffold by bringing the OTUB1 deubiquitinase close to SLC7A11, which will be deubiquitinated and stabilized.	[315]
circ0097009	miR-1261	Hepatocellular carcinoma Oncogene	It functions as a ceRNA by reducing the levels of miR-1261, which directly targets SLC7A11 3’UTR.	[316]
circEPSTI1	miR-375, miR-409-3p, and miR-515-5p	Cervical cancer Oncogene	It acts as a sponge of miR-375, miR-409-3p, and miR-515-5p, thus preventing them from reducing SLC7A11 levels.	[317]
lncP53RRA	p53	Lung cancer Tumor suppressor	It favours nuclear p53 by G3BP1 displacement and this leads to SLC7A11 downregulation.	[318]
lnc00618	LSH	Acute myeloid leukemia Tumor suppressor	It represses SLC7A11 through the inhibition of LSH, which is a positive transcription factor of SLC7A11 gene.	[319]
lncHEPFAL	unknown	Hepatocellular carcinoma Tumor suppressor	It may facilitate ubiquitination and degradation of SLC7A11.	[320]
lncPCAT1	c-Myc and miR-25-3p	Prostate cancer Oncogene	It directly increases c-Myc protein and sponges miR-25-3p thereby fosters SLC7A11 expression.	[321]
lncSNHG14	miR-206	Osteosarcoma Oncogene	It sequesters miR-206 that directly targets SLC7A11 with its consequent increase.	[322]
lncNRAV	miR-375-3p	Hepatocellular carcinoma Oncogene	It sponges miR-375-3p, thus attenuating SLC7A11 inhibition.	[323]
lncDUXAP8	SLC7A11	Hepatocellular carcinoma Oncogene	It directly promotes palmitoylation and stability of SLC7A11.	[324]
lncOIP5-AS1	miR-128-3p	Prostate cancer Oncogene	It functions as miR-128-3p sponge that directly targets the 3′ UTR of SLC7A11.	[325]
miR-375 miR-5096 miR-34c-3p miR-485-3p miR-27a	SLC7A11	Gastric carcinoma, Breast cancer, Oral squamos cell carcinoma, Hepatocellular Carcinoma, Pancreatic ductal adenocarcinoma, Non-small cell lung cancer cells Tumor suppressor	They bind the 3′ UTR of SLC7A11 mRNA**,** leading to its post-transcriptional repression.	[326,327,328,329,330]

**Table 4:** ncRNAs in the regulation of ferroptosis and of the p53-GPX4 axis in tumors.

ncRNA	Target	Cancer Type and Tumor Significance	Mechanism of Action on GPX4	Refs
circKDM4C	let-7b-5p	Acute myeloid leukemia Tumor suppressor	It sponges let-7b-5p that directly targets p53, thus enhancing ferroptosis associated to GPX4 downregulation.	[91]
circDTL	miR-1287-5p/GPX4 axis	Non-small cell lung cancer cells Oncogene	It sequesters miR-1287-5p that directly targets GPX4 with its consequent increase.	[331]
circOMA1	miR-145-5p	Prolactinoma Oncogene	It sponges miR-145-5p that directly targets GCLM provoking GPX4 increase.	[332]
circKIF4A	miR-1231	Thyroid cancer Oncogene	It sequesters miR-1231 that directly downregulates GPX4 expression, with its consequent increase.	[333]
circIL4R	miR-541-3p	Hepatocellular carcinoma Oncogene	It sponges miR-541-3p that directly targets GPX4 3’UTR, thus increasing its levels	[92]
circCDK14	miR-3938	Glioma Oncogene	It sequesters miR-3938 which directly decreases PDGFRA levels and indirectly increases GPX4 expression.	[313]
circPVT1	miR-30a-5p	Esophageal squamous cell carcinoma Oncogene	It sponges miR-30a-5p that negatively regulates FZD3 receptor, thus indirectly increasing GPX4 expression.	[314]
lncMACC1-AS1	STK33	Pancreatic ductal adenocarcinoma Oncogene	It directly binds and protects STK33 from degradation that consequently prevents GPX4 degradation.	[334]
lncHCG18	miR-450b-5p	Hepatocellular carcinoma Oncogene	It sequesters miR-450b-5p that directly decreases GPX4 transcripts and thus favoring GPX4 expression.	[335]
lncPVT1	miR-214-3p	Liver cancer Oncogene	It sponges miR-214-3p that directly targets GPX4 with its consequent increase	[336]
miR-4715-3p	AURKA	Gastric carcinoma Tumor suppressor	It directly targets AURKA which in turn maintains high levels of GPX4 to protect against ferroptosis.	[337]
miR-324-3p miR-1287-5p miR-15a miR-15a-3p miR-744-5p	GPX4	Lung cancer, Breast cancer, Osteosarcoma, Prostate cancer, Colorectal cancer, Non-small cell lung cancer cells Tumor suppressor	They directly bind the 3′UTR region of GPX4 mRNA, thus promoting ferroptosis.	[338,339,340,341,342,343]

## Data Availability

No new data were created or analyzed in this study. Data sharing is not applicable to this article.

## References

[1] Lei G., Zhuang L., Gan B. (2024). The Roles of Ferroptosis in Cancer: Tumor Suppression, Tumor Microenvironment, and Therapeutic Interventions. Cancer Cell.

[2] Chen S., Thorne R.F., Zhang X.D., Wu M., Liu L. (2021). Non-Coding RNAs, Guardians of the P53 Galaxy. Semin. Cancer Biol..

[3] Dixon S.J., Lemberg K.M., Lamprecht M.R., Skouta R., Zaitsev E.M., Gleason C.E., Patel D.N., Bauer A.J., Cantley A.M., Yang W.S. (2012). Ferroptosis: An Iron-Dependent Form of Nonapoptotic Cell Death. Cell.

[4] Gryzik M., Asperti M., Denardo A., Arosio P., Poli M. (2021). NCOA4-Mediated Ferritinophagy Promotes Ferroptosis Induced by Erastin, but Not by RSL3 in HeLa Cells. Biochim. Biophys. Acta Mol. Cell Res..

[5] Yan B., Ai Y., Sun Q., Ma Y., Cao Y., Wang J., Zhang Z., Wang X. (2021). Membrane Damage during Ferroptosis Is Caused by Oxidation of Phospholipids Catalyzed by the Oxidoreductases POR and CYB5R1. Mol. Cell.

[6] Yang W.-H., Huang Z., Wu J., Ding C.-K.C., Murphy S.K., Chi J.-T. (2020). A TAZ-ANGPTL4-NOX2 Axis Regulates Ferroptotic Cell Death and Chemoresistance in Epithelial Ovarian Cancer. Mol. Cancer Res..

[7] Jelinek A., Heyder L., Daude M., Plessner M., Krippner S., Grosse R., Diederich W.E., Culmsee C. (2018). Mitochondrial Rescue Prevents Glutathione Peroxidase-Dependent Ferroptosis. Free Radic. Biol. Med..

[8] Dixon S.J., Patel D.N., Welsch M., Skouta R., Lee E.D., Hayano M., Thomas A.G., Gleason C.E., Tatonetti N.P., Slusher B.S. (2014). Pharmacological Inhibition of Cystine-Glutamate Exchange Induces Endoplasmic Reticulum Stress and Ferroptosis. Elife.

[9] Zhang Y., Shi J., Liu X., Feng L., Gong Z., Koppula P., Sirohi K., Li X., Wei Y., Lee H. (2018). BAP1 Links Metabolic Regulation of Ferroptosis to Tumour Suppression. Nat. Cell Biol..

[10] Bersuker K., Hendricks J.M., Li Z., Magtanong L., Ford B., Tang P.H., Roberts M.A., Tong B., Maimone T.J., Zoncu R. (2019). The CoQ Oxidoreductase FSP1 Acts Parallel to GPX4 to Inhibit Ferroptosis. Nature.

[11] Yang W.S., SriRamaratnam R., Welsch M.E., Shimada K., Skouta R., Viswanathan V.S., Cheah J.H., Clemons P.A., Shamji A.F., Clish C.B. (2014). Regulation of Ferroptotic Cancer Cell Death by GPX4. Cell.

[12] Maio N., Rouault T.A. (2020). Outlining the Complex Pathway of Mammalian Fe-S Cluster Biogenesis. Trends Biochem. Sci..

[13] Alvarez S.W., Sviderskiy V.O., Terzi E.M., Papagiannakopoulos T., Moreira A.L., Adams S., Sabatini D.M., Birsoy K., Possemato R. (2017). NFS1 Undergoes Positive Selection in Lung Tumours and Protects Cells from Ferroptosis. Nature.

[14] Yang W.S., Kim K.J., Gaschler M.M., Patel M., Shchepinov M.S., Stockwell B.R. (2016). Peroxidation of Polyunsaturated Fatty Acids by Lipoxygenases Drives Ferroptosis. Proc. Natl. Acad. Sci. USA.

[15] Shah R., Shchepinov M.S., Pratt D.A. (2018). Resolving the Role of Lipoxygenases in the Initiation and Execution of Ferroptosis. ACS Cent. Sci..

[16] Zou Y., Li H., Graham E.T., Deik A.A., Eaton J.K., Wang W., Sandoval-Gomez G., Clish C.B., Doench J.G., Schreiber S.L. (2020). Cytochrome P450 Oxidoreductase Contributes to Phospholipid Peroxidation in Ferroptosis. Nat. Chem. Biol..

[17] Punziano C., Trombetti S., Cesaro E., Grosso M., Faraonio R. (2024). Antioxidant Systems as Modulators of Ferroptosis: Focus on Transcription Factors. Antioxidants.

[18] Lee J., Roh J.-L. (2025). Ferroptosis: Iron Release Mechanisms in the Bioenergetic Process. Cancer Metastasis Rev..

[19] Park M.W., Cha H.W., Kim J., Kim J.H., Yang H., Yoon S., Boonpraman N., Yi S.S., Yoo I.D., Moon J.-S. (2021). NOX4 Promotes Ferroptosis of Astrocytes by Oxidative Stress-Induced Lipid Peroxidation via the Impairment of Mitochondrial Metabolism in Alzheimer’s Diseases. Redox Biol..

[20] Liu Y., Lu S., Wu L.-L., Yang L., Yang L., Wang J. (2023). The Diversified Role of Mitochondria in Ferroptosis in Cancer Cell. Death Dis..

[21] Kim J.W., Lee J.-Y., Oh M., Lee E.-W. (2023). An Integrated View of Lipid Metabolism in Ferroptosis Revisited via Lipidomic Analysis. Exp. Mol. Med..

[22] Amoah A.-S., Pestov N.B., Korneenko T.V., Prokhorenko I.A., Kurakin G.F., Barlev N.A. (2024). Lipoxygenases at the Intersection of Infection and Carcinogenesis. Int. J. Mol. Sci..

[23] Li C., Zhang Y., Liu J., Kang R., Klionsky D.J., Tang D. (2021). Mitochondrial DNA Stress Triggers Autophagy-Dependent Ferroptotic Death. Autophagy.

[24] Shintoku R., Takigawa Y., Yamada K., Kubota C., Yoshimoto Y., Takeuchi T., Koshiishi I., Torii S. (2017). Lipoxygenase-Mediated Generation of Lipid Peroxides Enhances Ferroptosis Induced by Erastin and RSL3. Cancer Sci..

[25] Wenzel S.E., Tyurina Y.Y., Zhao J., St Croix C.M., Dar H.H., Mao G., Tyurin V.A., Anthonymuthu T.S., Kapralov A.A., Amoscato A.A. (2017). PEBP1 Wardens Ferroptosis by Enabling Lipoxygenase Generation of Lipid Death Signals. Cell.

[26] Ou Y., Wang S.-J., Li D., Chu B., Gu W. (2016). Activation of SAT1 Engages Polyamine Metabolism with P53-Mediated Ferroptotic Responses. Proc. Natl. Acad. Sci. USA.

[27] Chu B., Kon N., Chen D., Li T., Liu T., Jiang L., Song S., Tavana O., Gu W. (2019). ALOX12 Is Required for P53-Mediated Tumour Suppression through a Distinct Ferroptosis Pathway. Nat. Cell Biol..

[28] Jia R., Han J., Liu X., Li K., Lai W., Bian L., Yan J., Xi Z. (2023). Correction: Jia et al. Exposure to Polypropylene Microplastics via Oral Ingestion Induces Colonic Apoptosis and Intestinal Barrier Damage through Oxidative Stress and Inflammation in Mice. Toxics 2023, 11, 127. Toxics.

[29] Lagrost L., Masson D. (2022). The Expanding Role of Lyso-Phosphatidylcholine Acyltransferase-3 (LPCAT3), a Phospholipid Remodeling Enzyme, in Health and Disease. Curr. Opin. Lipidol.

[30] Cui J., Wang Y., Tian X., Miao Y., Ma L., Zhang C., Xu X., Wang J., Fang W., Zhang X. (2023). LPCAT3 Is Transcriptionally Regulated by YAP/ZEB/EP300 and Collaborates with ACSL4 and YAP to Determine Ferroptosis Sensitivity. Antioxid. Redox Signal..

[31] Doll S., Proneth B., Tyurina Y.Y., Panzilius E., Kobayashi S., Ingold I., Irmler M., Beckers J., Aichler M., Walch A. (2017). ACSL4 Dictates Ferroptosis Sensitivity by Shaping Cellular Lipid Composition. Nat. Chem. Biol..

[32] Tang D., Kroemer G. (2020). Peroxisome: The New Player in Ferroptosis. Signal Transduct. Target. Ther..

[33] Lee H., Zhuang L., Gan B. (2021). Ether Phospholipids Govern Ferroptosis. J. Genet. Genom..

[34] Cui W., Liu D., Gu W., Chu B. (2021). Peroxisome-Driven Ether-Linked Phospholipids Biosynthesis Is Essential for Ferroptosis. Cell Death Differ..

[35] Friedmann Angeli J.P., Schneider M., Proneth B., Tyurina Y.Y., Tyurin V.A., Hammond V.J., Herbach N., Aichler M., Walch A., Eggenhofer E. (2014). Inactivation of the Ferroptosis Regulator Gpx4 Triggers Acute Renal Failure in Mice. Nat. Cell Biol..

[36] Ingold I., Berndt C., Schmitt S., Doll S., Poschmann G., Buday K., Roveri A., Peng X., Porto Freitas F., Seibt T. (2018). Selenium Utilization by GPX4 Is Required to Prevent Hydroperoxide-Induced Ferroptosis. Cell.

[37] Murphy T.H., Miyamoto M., Sastre A., Schnaar R.L., Coyle J.T. (1989). Glutamate Toxicity in a Neuronal Cell Line Involves Inhibition of Cystine Transport Leading to Oxidative Stress. Neuron.

[38] Sato H., Tamba M., Ishii T., Bannai S. (1999). Cloning and Expression of a Plasma Membrane Cystine/Glutamate Exchange Transporter Composed of Two Distinct Proteins. J. Biol. Chem..

[39] Ursini F., Maiorino M. (2020). Lipid Peroxidation and Ferroptosis: The Role of GSH and GPx4. Free Radic. Biol. Med..

[40] Kennedy L., Sandhu J.K., Harper M.-E., Cuperlovic-Culf M. (2020). Role of Glutathione in Cancer: From Mechanisms to Therapies. Biomolecules.

[41] Harris I.S., DeNicola G.M. (2020). The Complex Interplay between Antioxidants and ROS in Cancer. Trends Cell Biol..

[42] Hider R., Aviles M.V., Chen Y.-L., Latunde-Dada G.O. (2021). The Role of GSH in Intracellular Iron Trafficking. Int. J. Mol. Sci..

[43] Patel S.J., Protchenko O., Shakoury-Elizeh M., Baratz E., Jadhav S., Philpott C.C. (2021). The Iron Chaperone and Nucleic Acid-Binding Activities of Poly(rC)-Binding Protein 1 Are Separable and Independently Essential. Proc. Natl. Acad. Sci. USA.

[44] Paulsen C.E., Carroll K.S. (2013). Cysteine-Mediated Redox Signaling: Chemistry, Biology, and Tools for Discovery. Chem. Rev..

[45] Daher B., Vučetić M., Pouysségur J. (2020). Cysteine Depletion, a Key Action to Challenge Cancer Cells to Ferroptotic Cell Death. Front. Oncol..

[46] Poltorack C.D., Dixon S.J. (2022). Understanding the Role of Cysteine in Ferroptosis: Progress & Paradoxes. FEBS. J.

[47] Hayano M., Yang W.S., Corn C.K., Pagano N.C., Stockwell B.R. (2016). Loss of Cysteinyl-tRNA Synthetase (CARS) Induces the Transsulfuration Pathway and Inhibits Ferroptosis Induced by Cystine Deprivation. Cell Death Differ..

[48] Wang L., Cai H., Hu Y., Liu F., Huang S., Zhou Y., Yu J., Xu J., Wu F. (2018). A Pharmacological Probe Identifies Cystathionine β-Synthase as a New Negative Regulator for Ferroptosis. Cell Death Dis..

[49] Shimada K., Skouta R., Kaplan A., Yang W.S., Hayano M., Dixon S.J., Brown L.M., Valenzuela C.A., Wolpaw A.J., Stockwell B.R. (2016). Global Survey of Cell Death Mechanisms Reveals Metabolic Regulation of Ferroptosis. Nat. Chem. Biol..

[50] Doll S., Freitas F.P., Shah R., Aldrovandi M., da Silva M.C., Ingold I., Goya Grocin A., Xavier da Silva T.N., Panzilius E., Scheel C.H. (2019). FSP1 Is a Glutathione-Independent Ferroptosis Suppressor. Nature.

[51] Mishima E., Ito J., Wu Z., Nakamura T., Wahida A., Doll S., Tonnus W., Nepachalovich P., Eggenhofer E., Aldrovandi M. (2022). A Non-Canonical Vitamin K Cycle Is a Potent Ferroptosis Suppressor. Nature.

[52] Lee J., Roh J.-L. (2023). Unleashing Ferroptosis in Human Cancers: Targeting Ferroptosis Suppressor Protein 1 for Overcoming Therapy Resistance. Antioxidants.

[53] Mao C., Liu X., Zhang Y., Lei G., Yan Y., Lee H., Koppula P., Wu S., Zhuang L., Fang B. (2021). DHODH-Mediated Ferroptosis Defence Is a Targetable Vulnerability in Cancer. Nature.

[54] Wu S., Mao C., Kondiparthi L., Poyurovsky M.V., Olszewski K., Gan B. (2022). A Ferroptosis Defense Mechanism Mediated by Glycerol-3-Phosphate Dehydrogenase 2 in Mitochondria. Proc. Natl. Acad. Sci. USA.

[55] Maiorino M., Conrad M., Ursini F. (2018). GPx4, Lipid Peroxidation, and Cell Death: Discoveries, Rediscoveries, and Open Issues. Antioxid. Redox Signal..

[56] Xie Y., Kang R., Klionsky D.J., Tang D. (2023). GPX4 in Cell Death, Autophagy, and Disease. Autophagy.

[57] Imai H., Hirao F., Sakamoto T., Sekine K., Mizukura Y., Saito M., Kitamoto T., Hayasaka M., Hanaoka K., Nakagawa Y. (2003). Early Embryonic Lethality Caused by Targeted Disruption of the Mouse PHGPx Gene. Biochem. Biophys. Res. Commun..

[58] Yant L.J., Ran Q., Rao L., Van Remmen H., Shibatani T., Belter J.G., Motta L., Richardson A., Prolla T.A. (2003). The Selenoprotein GPX4 Is Essential for Mouse Development and Protects from Radiation and Oxidative Damage Insults. Free Radic. Biol. Med..

[59] Garry M.R., Kavanagh T.J., Faustman E.M., Sidhu J.S., Liao R., Ware C., Vliet P.A., Deeb S.S. (2008). Sensitivity of Mouse Lung Fibroblasts Heterozygous for GPx4 to Oxidative Stress. Free Radic. Biol. Med..

[60] de Haan J.B., Bladier C., Lotfi-Miri M., Taylor J., Hutchinson P., Crack P.J., Hertzog P., Kola I. (2004). Fibroblasts Derived from Gpx1 Knockout Mice Display Senescent-like Features and Are Susceptible to H2O2-Mediated Cell Death. Free Radic. Biol. Med..

[61] Seiler A., Schneider M., Förster H., Roth S., Wirth E.K., Culmsee C., Plesnila N., Kremmer E., Rådmark O., Wurst W. (2008). Glutathione Peroxidase 4 Senses and Translates Oxidative Stress into 12/15-Lipoxygenase Dependent- and AIF-Mediated Cell Death. Cell Metab..

[62] Schnurr K., Belkner J., Ursini F., Schewe T., Kühn H. (1996). The Selenoenzyme Phospholipid Hydroperoxide Glutathione Peroxidase Controls the Activity of the 15-Lipoxygenase with Complex Substrates and Preserves the Specificity of the Oxygenation Products. J. Biol. Chem..

[63] Zuo S., Yu J., Pan H., Lu L. (2020). Novel Insights on Targeting Ferroptosis in Cancer Therapy. Biomark. Res..

[64] Sun S., Shen J., Jiang J., Wang F., Min J. (2023). Targeting Ferroptosis Opens New Avenues for the Development of Novel Therapeutics. Signal Transduct. Target. Ther..

[65] Wang X., Zhou Y., Min J., Wang F. (2023). Zooming in and out of Ferroptosis in Human Disease. Front. Med..

[66] Jiang X., Stockwell B.R., Conrad M. (2021). Ferroptosis: Mechanisms, Biology and Role in Disease. Nat. Rev. Mol. Cell Biol..

[67] Xu Y., Zhao J., Zhao Y., Zhou L., Qiao H., Xu Q., Liu Y. (2023). The Role of Ferroptosis in Neurodegenerative Diseases. Mol. Biol. Rep..

[68] Chen Z., Jiang J., Fu N., Chen L. (2022). Targetting Ferroptosis for Blood Cell-Related Diseases. J. Drug Target..

[69] Yang L., Liu Y., Zhou S., Feng Q., Lu Y., Liu D., Liu Z. (2023). Novel Insight into Ferroptosis in Kidney Diseases. Am. J. Nephrol..

[70] Fang X., Ardehali H., Min J., Wang F. (2023). The Molecular and Metabolic Landscape of Iron and Ferroptosis in Cardiovascular Disease. Nat. Rev. Cardiol..

[71] Coradduzza D., Congiargiu A., Chen Z., Zinellu A., Carru C., Medici S. (2023). Ferroptosis and Senescence: A Systematic Review. Int. J. Mol. Sci..

[72] Lei G., Zhuang L., Gan B. (2022). Targeting Ferroptosis as a Vulnerability in Cancer. Nat. Rev. Cancer.

[73] Dai E., Han L., Liu J., Xie Y., Zeh H.J., Kang R., Bai L., Tang D. (2020). Ferroptotic Damage Promotes Pancreatic Tumorigenesis through a TMEM173/STING-Dependent DNA Sensor Pathway. Nat. Commun..

[74] Ma X., Xiao L., Liu L., Ye L., Su P., Bi E., Wang Q., Yang M., Qian J., Yi Q. (2021). CD36-Mediated Ferroptosis Dampens Intratumoral CD8+ T Cell Effector Function and Impairs Their Antitumor Ability. Cell Metab..

[75] Chen Z., Wang W., Abdul Razak S.R., Han T., Ahmad N.H., Li X. (2023). Ferroptosis as a Potential Target for Cancer Therapy. Cell Death Dis..

[76] Zuo Y.-B., Zhang Y.-F., Zhang R., Tian J.-W., Lv X.-B., Li R., Li S.-P., Cheng M.-D., Shan J., Zhao Z. (2022). Ferroptosis in Cancer Progression: Role of Noncoding RNAs. Int. J. Biol. Sci..

[77] Gong H., Li Z., Wu Z., Lian G., Su Z. (2024). Modulation of Ferroptosis by Non-coding RNAs in Cancers: Potential Biomarkers for Cancer Diagnose and Therapy. Pathol. Res. Pract..

[78] Zhang Q., Fan X., Zhang X., Ju S. (2023). Ferroptosis in Tumors and Its Relationship to Other Programmed Cell Death: Role of Non-Coding RNAs. J. Transl. Med..

[79] Ni W.-J., Leng X.-M. (2016). miRNA-Dependent Activation of mRNA Translation. Microrna.

[80] Ramchandran R., Chaluvally-Raghavan P. (2017). miRNA-Mediated RNA Activation in Mammalian Cells. Adv. Exp. Med. Biol..

[81] Oliveto S., Mancino M., Manfrini N., Biffo S. (2017). Role of microRNAs in Translation Regulation and Cancer. World J. Biol. Chem..

[82] Liccardo R., Sessa R., Trombetti S., De Rosa M., Izzo P., Grosso M., Duraturo F. (2021). MiR-137 Targets the 3’ Untranslated Region of MSH2: Potential Implications in Lynch Syndrome-Related Colorectal Cancer. Cancers.

[83] Sessa R., Trombetti S., Bianco A.L., Amendola G., Catapano R., Cesaro E., Petruzziello F., D’Armiento M., Maruotti G.M., Menna G. (2024). miR-1202 Acts as Anti-oncomiR in Myeloid Leukaemia by down-Modulating GATA-1S Expression. Open Biol..

[84] Mattick J.S., Amaral P.P., Carninci P., Carpenter S., Chang H.Y., Chen L.-L., Chen R., Dean C., Dinger M.E., Fitzgerald K.A. (2023). Long Non-Coding RNAs: Definitions, Functions, Challenges and Recommendations. Nat. Rev. Mol. Cell Biol..

[85] Ferrer J., Dimitrova N. (2024). Transcription Regulation by Long Non-Coding RNAs: Mechanisms and Disease Relevance. Nat. Rev. Mol. Cell Biol..

[86] Statello L., Guo C.-J., Chen L.-L., Huarte M. (2021). Gene Regulation by Long Non-Coding RNAs and Its Biological Functions. Nat. Rev. Mol. Cell Biol..

[87] Godet A.-C., Roussel E., Laugero N., Morfoisse F., Lacazette E., Garmy-Susini B., Prats A.-C. (2024). Translational Control by Long Non-Coding RNAs. Biochimie.

[88] Wei C., Xu Y., Shen Q., Li R., Xiao X., Saw P.E., Xu X. (2023). Role of Long Non-Coding RNAs in Cancer: From Subcellular Localization to Nanoparticle-Mediated Targeted Regulation. Mol. Ther. Nucleic Acids.

[89] Santer L., Bär C., Thum T. (2019). Circular RNAs: A Novel Class of Functional RNA Molecules with a Therapeutic Perspective. Mol. Ther..

[90] Liu R., Zhou Y., Cao Y. (2023). CircRNA and Ferroptosis in Human Disease: Insights for New Treatments. Anim. Model. Exp. Med..

[91] Dong L.-H., Huang J.-J., Zu P., Liu J., Gao X., Du J.-W., Li Y.-F. (2021). CircKDM4C Upregulates P53 by Sponging Hsa-Let-7b-5p to Induce Ferroptosis in Acute Myeloid Leukemia. Environ. Toxicol..

[92] Xu Q., Zhou L., Yang G., Meng F., Wan Y., Wang L., Zhang L. (2020). CircIL4R Facilitates the Tumorigenesis and Inhibits Ferroptosis in Hepatocellular Carcinoma by Regulating the miR-541-3p/GPX4 Axis. Cell Biol. Int..

[93] Liu Z., Wang Q., Wang X., Xu Z., Wei X., Li J. (2020). Circular RNA cIARS Regulates Ferroptosis in HCC Cells through Interacting with RNA Binding Protein ALKBH5. Cell Death Discov..

[94] Levine A.J. (2020). P53: 800 Million Years of Evolution and 40 Years of Discovery. Nat. Rev. Cancer.

[95] Liu Y., Su Z., Tavana O., Gu W. (2024). Understanding the Complexity of P53 in a New Era of Tumor Suppression. Cancer Cell.

[96] Lahav G., Rosenfeld N., Sigal A., Geva-Zatorsky N., Levine A.J., Elowitz M.B., Alon U. (2004). Dynamics of the P53-Mdm2 Feedback Loop in Individual Cells. Nat. Genet..

[97] Haupt Y., Maya R., Kazaz A., Oren M. (1997). Mdm2 Promotes the Rapid Degradation of P53. Nature.

[98] Weinberg R.L., Freund S.M.V., Veprintsev D.B., Bycroft M., Fersht A.R. (2004). Regulation of DNA Binding of P53 by Its C-Terminal Domain. J. Mol. Biol..

[99] Pflaum J., Schlosser S., MÃ¼ller M. (2014). P53 Family and Cellular Stress Responses in Cancer. Front. Oncol..

[100] Feroz W., Sheikh A.M.A. (2020). Exploring the Multiple Roles of Guardian of the Genome: P53. Egypt. J. Med. Hum. Genet..

[101] Engeland K. (2022). Cell Cycle Regulation: P53-P21-RB Signaling. Cell Death Differ..

[102] Uxa S., Bernhart S.H., Mages C.F.S., Fischer M., Kohler R., Hoffmann S., Stadler P.F., Engeland K., Müller G.A. (2019). DREAM and RB Cooperate to Induce Gene Repression and Cell-Cycle Arrest in Response to P53 Activation. Nucleic Acids Res..

[103] Sadasivam S., DeCaprio J.A. (2013). The DREAM Complex: Master Coordinator of Cell Cycle-Dependent Gene Expression. Nat. Rev. Cancer.

[104] Engeland K. (2018). Cell Cycle Arrest through Indirect Transcriptional Repression by P53: I Have a DREAM. Cell Death Differ..

[105] Fischer M., Grossmann P., Padi M., DeCaprio J.A. (2016). Integration of TP53, DREAM, MMB-FOXM1 and RB-E2F Target Gene Analyses Identifies Cell Cycle Gene Regulatory Networks. Nucleic Acids Res..

[106] Grossman S.R. (2001). P300/CBP/P53 Interaction and Regulation of the P53 Response. Eur. J. Biochem..

[107] Hengstermann A., Linares L.K., Ciechanover A., Whitaker N.J., Scheffner M. (2001). Complete Switch from Mdm2 to Human Papillomavirus E6-Mediated Degradation of P53 in Cervical Cancer Cells. Proc. Natl. Acad. Sci. USA.

[108] Lee C., Cho Y. (2002). Interactions of SV40 Large T Antigen and Other Viral Proteins with Retinoblastoma Tumour Suppressor. Rev. Med. Virol..

[109] Serrano M., Lin A.W., McCurrach M.E., Beach D., Lowe S.W. (1997). Oncogenic Ras Provokes Premature Cell Senescence Associated with Accumulation of P53 and p16INK4a. Cell.

[110] Rufini A., Tucci P., Celardo I., Melino G. (2013). Senescence and Aging: The Critical Roles of P53. Oncogene.

[111] Liao Z., Yeo H.L., Wong S.W., Zhao Y. (2021). Cellular Senescence: Mechanisms and Therapeutic Potential. Biomedicines.

[112] Ngoi N.Y., Liew A.Q., Chong S.J.F., Davids M.S., Clement M.-V., Pervaiz S. (2021). The Redox-Senescence Axis and Its Therapeutic Targeting. Redox Biol..

[113] Lettieri-Barbato D., Aquilano K., Punziano C., Minopoli G., Faraonio R. (2022). MicroRNAs, Long Non-Coding RNAs, and Circular RNAs in the Redox Control of Cell Senescence. Antioxidants.

[114] Yang J., Xu Z.-P., Huang Y., Hamrick H.E., Duerksen-Hughes P.J., Yu Y.-N. (2004). ATM and ATR: Sensing DNA Damage. World. J. Gastroenterol..

[115] Chen Z., Trotman L.C., Shaffer D., Lin H.-K., Dotan Z.A., Niki M., Koutcher J.A., Scher H.I., Ludwig T., Gerald W. (2005). Crucial Role of P53-Dependent Cellular Senescence in Suppression of Pten-Deficient Tumorigenesis. Nature.

[116] Collado M., Serrano M. (2010). Senescence in Tumours: Evidence from Mice and Humans. Nat. Rev. Cancer.

[117] Debacq-Chainiaux F., Borlon C., Pascal T., Royer V., Eliaers F., Ninane N., Carrard G., Friguet B., De Longueville F., Boffe S. (2005). Repeated Exposure of Human Skin Fibroblasts to UVB at Subcytotoxic Level Triggers Premature Senescence through the TGF-Β1 Signaling Pathway. J. Cell Sci..

[118] Coppé J.-P., Patil C.K., Rodier F., Sun Y., Muñoz D.P., Goldstein J., Nelson P.S., Desprez P.-Y., Campisi J. (2008). Senescence-Associated Secretory Phenotypes Reveal Cell-Nonautonomous Functions of Oncogenic RAS and the P53 Tumor Suppressor. PLoS Biol..

[119] Mijit M., Caracciolo V., Melillo A., Amicarelli F., Giordano A. (2020). Role of P53 in the Regulation of Cellular Senescence. Biomolecules.

[120] Gao M., Li H., Zhang J. (2025). RB Functions as a Key Regulator of Senescence and Tumor Suppression. Semin. Cancer Biol..

[121] Pawge G., Khatik G.L. (2021). P53 Regulated Senescence Mechanism and Role of Its Modulators in Age-Related Disorders. Biochem. Pharmacol..

[122] Abuetabh Y., Wu H.H., Chai C., Al Yousef H., Persad S., Sergi C.M., Leng R. (2022). DNA Damage Response Revisited: The P53 Family and Its Regulators Provide Endless Cancer Therapy Opportunities. Exp. Mol. Med..

[123] Yosef R., Pilpel N., Papismadov N., Gal H., Ovadya Y., Vadai E., Miller S., Porat Z., Ben-Dor S., Krizhanovsky V. (2017). P21 Maintains Senescent Cell Viability under Persistent DNA Damage Response by Restraining JNK and Caspase Signaling. EMBO J..

[124] Tarangelo A., Magtanong L., Bieging-Rolett K.T., Li Y., Ye J., Attardi L.D., Dixon S.J. (2018). P53 Suppresses Metabolic Stress-Induced Ferroptosis in Cancer Cells. Cell Rep..

[125] Rana T., Jiang C., Banerjee S., Yi N., Zmijewski J.W., Liu G., Liu R.-M. (2023). PAI-1 Regulation of P53 Expression and Senescence in Type II Alveolar Epithelial Cells. Cells.

[126] Kortlever R.M., Higgins P.J., Bernards R. (2006). Plasminogen Activator Inhibitor-1 Is a Critical Downstream Target of P53 in the Induction of Replicative Senescence. Nat. Cell Biol..

[127] Kawarada Y., Inoue Y., Kawasaki F., Fukuura K., Sato K., Tanaka T., Itoh Y., Hayashi H. (2016). TGF-β Induces P53/Smads Complex Formation in the PAI-1 Promoter to Activate Transcription. Sci. Rep..

[128] Kortlever R.M., Nijwening J.H., Bernards R. (2008). Transforming Growth Factor-β Requires Its Target Plasminogen Activator Inhibitor-1 for Cytostatic Activity. J. Biol. Chem..

[129] Volonte D., Zhang K., Lisanti M.P., Galbiati F. (2002). Expression of Caveolin-1 Induces Premature Cellular Senescence in Primary Cultures of Murine Fibroblasts: Stress-Induced Premature Senescence Upregulates the Expression of Endogenous Caveolin-1. MBoC.

[130] Volonte D., Galbiati F. (2011). Polymerase I and Transcript Release Factor (PTRF)/Cavin-1 Is a Novel Regulator of Stress-Induced Premature Senescence. J. Biol. Chem..

[131] Samarakoon R., Higgins S.P., Higgins C.E., Higgins P.J. (2019). The TGF-Β1/P53/PAI-1 Signaling Axis in Vascular Senescence: Role of Caveolin-1. Biomolecules.

[132] Bartholomew J.N., Volonte D., Galbiati F. (2009). Caveolin-1 Regulates the Antagonistic Pleiotropic Properties of Cellular Senescence through a Novel Mdm2/P53-Mediated Pathway. Cancer Res..

[133] Feng Y., Wei H., Lyu M., Yu Z., Chen J., Lyu X., Zhuang F. (2024). Iron Retardation in Lysosomes Protects Senescent Cells from Ferroptosis. Aging.

[134] Oda E., Ohki R., Murasawa H., Nemoto J., Shibue T., Yamashita T., Tokino T., Taniguchi T., Tanaka N. (2000). Noxa, a BH3-Only Member of the Bcl-2 Family and Candidate Mediator of P53-Induced Apoptosis. Science.

[135] Yu J., Zhang L., Hwang P.M., Kinzler K.W., Vogelstein B. (2001). PUMA Induces the Rapid Apoptosis of Colorectal Cancer Cells. Mol. Cell.

[136] Nakano K., Vousden K.H. (2001). PUMA, a Novel Proapoptotic Gene, Is Induced by P53. Mol. Cell.

[137] Han J., Flemington C., Houghton A.B., Gu Z., Zambetti G.P., Lutz R.J., Zhu L., Chittenden T. (2001). Expression of Bbc3, a pro-Apoptotic BH3-Only Gene, Is Regulated by Diverse Cell Death and Survival Signals. Proc. Natl. Acad. Sci. USA.

[138] Michalak E.M., Villunger A., Adams J.M., Strasser A. (2008). In Several Cell Types Tumour Suppressor P53 Induces Apoptosis Largely via Puma but Noxa Can Contribute. Cell Death Differ..

[139] Tsujimoto Y. (2003). Cell Death Regulation by the Bcl-2 Protein Family in the Mitochondria. J. Cell. Physiol..

[140] Letai A., Bassik M.C., Walensky L.D., Sorcinelli M.D., Weiler S., Korsmeyer S.J. (2002). Distinct BH3 Domains Either Sensitize or Activate Mitochondrial Apoptosis, Serving as Prototype Cancer Therapeutics. Cancer Cell.

[141] Marchenko N.D., Moll U.M. (2014). Mitochondrial Death Functions of P53. Mol. Cell. Oncol..

[142] Jiang L., Kon N., Li T., Wang S.-J., Su T., Hibshoosh H., Baer R., Gu W. (2015). Ferroptosis as a P53-Mediated Activity during Tumour Suppression. Nature.

[143] Venkatesh D., Stockwell B.R., Prives C. (2020). P21 Can Be a Barrier to Ferroptosis Independent of P53. Aging.

[144] Liu J., Zhang C., Wang J., Hu W., Feng Z. (2020). The Regulation of Ferroptosis by Tumor Suppressor P53 and Its Pathway. Int. J. Mol. Sci..

[145] Weizer-Stern O., Adamsky K., Margalit O., Ashur-Fabian O., Givol D., Amariglio N., Rechavi G. (2007). Hepcidin, a Key Regulator of Iron Metabolism, Is Transcriptionally Activated by P53. Br. J. Haematol..

[146] Funauchi Y., Tanikawa C., Yi Lo P.H., Mori J., Daigo Y., Takano A., Miyagi Y., Okawa A., Nakamura Y., Matsuda K. (2015). Regulation of Iron Homeostasis by the P53-ISCU Pathway. Sci. Rep..

[147] Zhao N., Zhang A.-S., Wortham A.M., Jue S., Knutson M.D., Enns C.A. (2017). The Tumor Suppressor, P53, Decreases the Metal Transporter, ZIP14. Nutrients.

[148] Zhang Y., Qian Y., Zhang J., Yan W., Jung Y.-S., Chen M., Huang E., Lloyd K., Duan Y., Wang J. (2017). Ferredoxin Reductase Is Critical for P53-Dependent Tumor Suppression via Iron Regulatory Protein 2. Genes Dev..

[149] Shimizu R., Lan N.N., Tai T.T., Adachi Y., Kawazoe A., Mu A., Taketani S. (2014). P53 Directly Regulates the Transcription of the Human Frataxin Gene and Its Lack of Regulation in Tumor Cells Decreases the Utilization of Mitochondrial Iron. Gene.

[150] Zhang Z., Guo M., Shen M., Kong D., Zhang F., Shao J., Tan S., Wang S., Chen A., Cao P. (2020). The BRD7-P53-SLC25A28 Axis Regulates Ferroptosis in Hepatic Stellate Cells. Redox Biol..

[151] Shen J., Sheng X., Chang Z., Wu Q., Wang S., Xuan Z., Li D., Wu Y., Shang Y., Kong X. (2014). Iron Metabolism Regulates P53 Signaling through Direct Heme-P53 Interaction and Modulation of P53 Localization, Stability, and Function. Cell Rep..

[152] Liang S.X., Richardson D.R. (2003). The Effect of Potent Iron Chelators on the Regulation of P53: Examination of the Expression, Localization and DNA-Binding Activity of P53 and the Transactivation of WAF1. Carcinogenesis.

[153] Kim B.M., Choi J.Y., Kim Y.J., Woo H.D., Chung H.W. (2007). Desferrioxamine (DFX) Has Genotoxic Effects on Cultured Human Lymphocytes and Induces the P53-Mediated Damage Response. Toxicology.

[154] Nemeth E., Tuttle M.S., Powelson J., Vaughn M.B., Donovan A., Ward D.M., Ganz T., Kaplan J. (2004). Hepcidin Regulates Cellular Iron Efflux by Binding to Ferroportin and Inducing Its Internalization. Science.

[155] Yilmaz D., Tharehalli U., Paganoni R., Knoop P., Gruber A., Chen Y., Dong R., Leithäuser F., Seufferlein T., Leopold K. (2025). Iron Metabolism in a Mouse Model of Hepatocellular Carcinoma. Sci. Rep..

[156] Torti S.V., Torti F.M. (2013). Iron and Cancer: More Ore to Be Mined. Nat. Rev. Cancer.

[157] Torti S.V., Torti F.M. (2020). Iron: The Cancer Connection. Mol. Aspects Med..

[158] Hann H.W., Stahlhut M.W., Blumberg B.S. (1988). Iron Nutrition and Tumor Growth: Decreased Tumor Growth in Iron-Deficient Mice. Cancer Res..

[159] Zhang J., Chen X. (2019). P53 Tumor Suppressor and Iron Homeostasis. FEBS J..

[160] Liu B., Chen Y., St Clair D.K. (2008). ROS and P53: A Versatile Partnership. Free Radic. Biol. Med..

[161] Sablina A.A., Budanov A.V., Ilyinskaya G.V., Agapova L.S., Kravchenko J.E., Chumakov P.M. (2005). The Antioxidant Function of the P53 Tumor Suppressor. Nat. Med..

[162] Bykov V.J.N., Lambert J.M.R., Hainaut P., Wiman K.G. (2009). Mutant P53 Rescue and Modulation of P53 Redox State. Cell Cycle.

[163] Eriksson S.E., Ceder S., Bykov V.J.N., Wiman K.G. (2019). P53 as a Hub in Cellular Redox Regulation and Therapeutic Target in Cancer. J. Mol. Cell Biol..

[164] May P., May E. (1999). Twenty Years of P53 Research: Structural and Functional Aspects of the P53 Protein. Oncogene.

[165] Rohaly G., Chemnitz J., Dehde S., Nunez A.M., Heukeshoven J., Deppert W., Dornreiter I. (2005). A Novel Human P53 Isoform Is an Essential Element of the ATR-Intra-S Phase Checkpoint. Cell.

[166] Wang H., Luo K., Tan L.-Z., Ren B.-G., Gu L.-Q., Michalopoulos G., Luo J.-H., Yu Y.P. (2012). P53-Induced Gene 3 Mediates Cell Death Induced by Glutathione Peroxidase 3. J. Biol. Chem..

[167] Kang M.Y., Kim H.-B., Piao C., Lee K.H., Hyun J.W., Chang I.-Y., You H.J. (2013). The Critical Role of Catalase in Prooxidant and Antioxidant Function of P53. Cell Death Differ..

[168] Matoba S., Kang J.-G., Patino W.D., Wragg A., Boehm M., Gavrilova O., Hurley P.J., Bunz F., Hwang P.M. (2006). P53 Regulates Mitochondrial Respiration. Science.

[169] Leary S.C., Sasarman F., Nishimura T., Shoubridge E.A. (2009). Human SCO2 Is Required for the Synthesis of CO II and as a Thiol-Disulphide Oxidoreductase for SCO1. Hum. Mol. Genet..

[170] Madan E., Gogna R., Kuppusamy P., Bhatt M., Mahdi A.A., Pati U. (2013). SCO2 Induces P53-Mediated Apoptosis by Thr845 Phosphorylation of ASK-1 and Dissociation of the ASK-1-Trx Complex. Mol. Cell. Biol..

[171] Gao M., Monian P., Quadri N., Ramasamy R., Jiang X. (2015). Glutaminolysis and Transferrin Regulate Ferroptosis. Mol. Cell.

[172] Suzuki S., Venkatesh D., Kanda H., Nakayama A., Hosokawa H., Lee E., Miki T., Stockwell B.R., Yokote K., Tanaka T. (2022). GLS2 Is a Tumor Suppressor and a Regulator of Ferroptosis in Hepatocellular Carcinoma. Cancer Res..

[173] Suzuki S., Tanaka T., Poyurovsky M.V., Nagano H., Mayama T., Ohkubo S., Lokshin M., Hosokawa H., Nakayama T., Suzuki Y. (2010). Phosphate-Activated Glutaminase (GLS2), a P53-Inducible Regulator of Glutamine Metabolism and Reactive Oxygen Species. Proc. Natl. Acad. Sci. USA.

[174] Matés J.M., Campos-Sandoval J.A., de Los Santos-Jiménez J., Segura J.A., Alonso F.J., Márquez J. (2020). Metabolic Reprogramming of Cancer by Chemicals That Target Glutaminase Isoenzymes. Curr. Med. Chem.

[175] Jennis M., Kung C.-P., Basu S., Budina-Kolomets A., Leu J.I.-J., Khaku S., Scott J.P., Cai K.Q., Campbell M.R., Porter D.K. (2016). An African-Specific Polymorphism in the TP53 Gene Impairs P53 Tumor Suppressor Function in a Mouse Model. Genes Dev..

[176] Jiang P., Du W., Wang X., Mancuso A., Gao X., Wu M., Yang X. (2011). P53 Regulates Biosynthesis through Direct Inactivation of Glucose-6-Phosphate Dehydrogenase. Nat. Cell Biol..

[177] Xu R., Wang W., Zhang W. (2023). Ferroptosis and the Bidirectional Regulatory Factor P53. Cell Death Discov..

[178] Saint-Germain E., Mignacca L., Vernier M., Bobbala D., Ilangumaran S., Ferbeyre G. (2017). SOCS1 Regulates Senescence and Ferroptosis by Modulating the Expression of P53 Target Genes. Aging.

[179] Yan Y., Teng H., Hang Q., Kondiparthi L., Lei G., Horbath A., Liu X., Mao C., Wu S., Zhuang L. (2023). SLC7A11 Expression Level Dictates Differential Responses to Oxidative Stress in Cancer Cells. Nat. Commun..

[180] Cramer S.L., Saha A., Liu J., Tadi S., Tiziani S., Yan W., Triplett K., Lamb C., Alters S.E., Rowlinson S. (2017). Systemic Depletion of L-Cyst(e)Ine with Cyst(e)Inase Increases Reactive Oxygen Species and Suppresses Tumor Growth. Nat. Med..

[181] Li X., Xiong W., Wang Y., Li Y., Cheng X., Liu W. (2023). P53 Activates the Lipoxygenase Activity of ALOX15B via Inhibiting SLC7A11 to Induce Ferroptosis in Bladder Cancer Cells. Lab. Investig..

[182] Koppula P., Zhang Y., Zhuang L., Gan B. (2018). Amino Acid Transporter SLC7A11/xCT at the Crossroads of Regulating Redox Homeostasis and Nutrient Dependency of Cancer. Cancer Commun..

[183] Sun L.-Y., Ke S.-B., Li B.-X., Chen F.-S., Huang Z.-Q., Li L., Zhang J.-F., Cai Y.-X., Zhu H.-J., Zhang X.-D. (2025). ANP32E Promotes Esophageal Cancer Progression and Paclitaxel Resistance via P53/SLC7A11 Axis-Regulated Ferroptosis. Int. Immunopharmacol..

[184] Hur W., Rhim H., Jung C.K., Kim J.D., Bae S.H., Jang J.W., Yang J.M., Oh S.-T., Kim D.G., Wang H.J. (2010). SOX4 Overexpression Regulates the P53-Mediated Apoptosis in Hepatocellular Carcinoma: Clinical Implication and Functional Analysis In Vitro. Carcinogenesis.

[185] Zhu H., Xu S. (2024). SOX4 Inhibits Ferroptosis and Promotes Proliferation of Endometrial Cancer Cells via the P53/SLC7A11 Signaling. J. Obstet. Gynaecol. Res..

[186] Yan X., Li Z., Chen H., Yang F., Tian Q., Zhang Y. (2024). Photodynamic Therapy Inhibits Cancer Progression and Induces Ferroptosis and Apoptosis by Targeting P53/GPX4/SLC7A11 Signaling Pathways in Cholangiocarcinoma. Photodiagnosis Photodyn. Ther..

[187] Xia M., Wu Y., Zhu H., Duan W. (2023). Tanshinone I Induces Ferroptosis in Gastric Cancer Cells via the KDM4D/P53 Pathway. Hum. Exp. Toxicol..

[188] Wu Y., Pi D., Zhou S., Yi Z., Dong Y., Wang W., Ye H., Chen Y., Zuo Q., Ouyang M. (2023). Ginsenoside Rh3 Induces Pyroptosis and Ferroptosis through the Stat3/P53/NRF2 Axis in Colorectal Cancer Cells. Acta Biochim. Biophys. Sin..

[189] Ming T., Lei J., Peng Y., Wang M., Liang Y., Tang S., Tao Q., Wang M., Tang X., He Z. (2024). Curcumin Suppresses Colorectal Cancer by Induction of Ferroptosis via Regulation of P53 and Solute Carrier Family 7 Member 11/Glutathione/Glutathione Peroxidase 4 Signaling Axis. Phytother. Res..

[190] Mao C., Gong L., Kang W. (2024). Effect and Mechanism of Resveratrol on Ferroptosis Mediated by P53/SLC7A11 in Oral Squamous Cell Carcinoma. BMC Oral Health.

[191] He D., Tan X.-N., Li L.-P., Gao W.-H., Tian X.-F., Zeng P.-H. (2024). Brazilin Actuates Ferroptosis in Breast Cancer Cells via P53/SLC7A11/GPX4 Signaling Pathway. Chin. J. Integr. Med..

[192] Zhang W.J., Hu C.L., Guo B.L., Liang X.P., Wang C.Y., Yang T. (2024). STAT5B Suppresses Ferroptosis by Promoting DCAF13 Transcription to Regulate P53/xCT Pathway to Promote Mantle Cell Lymphoma Progression. Biologics.

[193] Lei M., Zhang Y.-L., Huang F.-Y., Chen H.-Y., Chen M.-H., Wu R.-H., Dai S.-Z., He G.-S., Tan G.-H., Zheng W.-P. (2023). Gankyrin Inhibits Ferroptosis through the P53/SLC7A11/GPX4 Axis in Triple-Negative Breast Cancer Cells. Sci. Rep..

[194] Wang M., Mao C., Ouyang L., Liu Y., Lai W., Liu N., Shi Y., Chen L., Xiao D., Yu F. (2019). Long Noncoding RNA LINC00336 Inhibits Ferroptosis in Lung Cancer by Functioning as a Competing Endogenous RNA. Cell Death Differ..

[195] Cao J., Chen X., Jiang L., Lu B., Yuan M., Zhu D., Zhu H., He Q., Yang B., Ying M. (2020). DJ-1 Suppresses Ferroptosis through Preserving the Activity of S-Adenosyl Homocysteine Hydrolase. Nat. Commun..

[196] Kato I., Maita H., Takahashi-Niki K., Saito Y., Noguchi N., Iguchi-Ariga S.M.M., Ariga H. (2013). Oxidized DJ-1 Inhibits P53 by Sequestering P53 from Promoters in a DNA-Binding Affinity-Dependent Manner. Mol. Cell. Biol..

[197] Lee J.-Y., Kim W.K., Bae K.-H., Lee S.C., Lee E.-W. (2021). Lipid Metabolism and Ferroptosis. Biology.

[198] Mortensen M.S., Ruiz J., Watts J.L. (2023). Polyunsaturated Fatty Acids Drive Lipid Peroxidation during Ferroptosis. Cells.

[199] Gaschler M.M., Stockwell B.R. (2017). Lipid Peroxidation in Cell Death. Biochem. Biophys. Res. Commun..

[200] Wang B., Wang Y., Zhang J., Hu C., Jiang J., Li Y., Peng Z. (2023). ROS-Induced Lipid Peroxidation Modulates Cell Death Outcome: Mechanisms behind Apoptosis, Autophagy, and Ferroptosis. Arch. Toxicol..

[201] Qian B., Che L., Du Z.-B., Guo N.-J., Wu X.-M., Yang L., Zheng Z.-X., Gao Y.-L., Wang M.-Z., Chen X.-X. (2023). Protein Phosphatase 2A-B55β Mediated Mitochondrial p-GPX4 Dephosphorylation Promoted Sorafenib-Induced Ferroptosis in Hepatocellular Carcinoma via Regulating P53 Retrograde Signaling. Theranostics.

[202] Li Q.-Q., Li Q., Jia J.-N., Liu Z.-Q., Zhou H.-H., Mao X.-Y. (2018). 12/15 Lipoxygenase: A Crucial Enzyme in Diverse Types of Cell Death. Neurochem. Int..

[203] Yang X., Liu J., Wang C., Cheng K.K.-Y., Xu H., Li Q., Hua T., Jiang X., Sheng L., Mao J. (2021). miR-18a Promotes Glioblastoma Development by down-Regulating ALOXE3-Mediated Ferroptotic and Anti-Migration Activities. Oncogenesis.

[204] Ma X., Gan Y., Mai Z., Song Y., Zhang M., Xia W. (2025). Silencing HEATR1 Rescues Cisplatin Resistance of Non-Small Cell Lung Cancer by Inducing Ferroptosis via the P53/SAT1/ALOX15 Axis. Curr. Cancer Drug Targets.

[205] Gilbert B., Ahmad K., Roos J., Lehmann C., Chiba T., Ulrich-Rückert S., Smeenk L., van Heeringen S., Maier T.J., Groner B. (2015). 5-Lipoxygenase Is a Direct P53 Target Gene in Humans. Biochim. Biophys. Acta.

[206] Catalano A., Rodilossi S., Caprari P., Coppola V., Procopio A. (2005). 5-Lipoxygenase Regulates Senescence-like Growth Arrest by Promoting ROS-Dependent P53 Activation. EMBO J..

[207] Yang W.S., Stockwell B.R. (2016). Ferroptosis: Death by Lipid Peroxidation. Trends Cell Biol..

[208] Dixon S.J., Pratt D.A. (2023). Ferroptosis: A Flexible Constellation of Related Biochemical Mechanisms. Mol. Cell.

[209] Li G., Zhang H., Lai H., Liang G., Huang J., Zhao F., Xie X., Peng C. (2023). Erianin: A Phytoestrogen with Therapeutic Potential. Front. Pharmacol..

[210] Shen H., Geng Z., Nie X., Liu T. (2023). Erianin Induces Ferroptosis of Renal Cancer Stem Cells via Promoting ALOX12/P53 mRNA N6-Methyladenosine Modification. J. Cancer.

[211] Mandal S., Mandal A., Park M.H. (2015). Depletion of the Polyamines Spermidine and Spermine by Overexpression of Spermidine/Spermine N^1^-Acetyltransferase 1 (SAT1) Leads to Mitochondria-Mediated Apoptosis in Mammalian Cells. Biochem. J..

[212] Zhao G., Liang J., Zhang Y., Shan G., Bian Y., Gu J., Zhan C., Ge D. (2024). MNT Inhibits Lung Adenocarcinoma Ferroptosis and Chemosensitivity by Suppressing SAT1. Commun. Biol..

[213] Zhang F., Wang W., Tsuji Y., Torti S.V., Torti F.M. (2008). Post-Transcriptional Modulation of Iron Homeostasis during P53-Dependent Growth Arrest. J. Biol. Chem..

[214] Liuzzi J.P., Aydemir F., Nam H., Knutson M.D., Cousins R.J. (2006). Zip14 (Slc39a14) Mediates Non-Transferrin-Bound Iron Uptake into Cells. Proc. Natl. Acad. Sci. USA.

[215] Deng Z., Zeng W., Gao Y., Yang Z., Luo X., Li X., Sun G., Xiong E., Huang F., Luo G. (2025). Mesenchymal Stem Cells Prevent SLC39A14-Dependent Hepatocyte Ferroptosis through Exosomal miR-16-5p in Liver Graft. Adv. Sci..

[216] Shi Y., Ghosh M., Kovtunovych G., Crooks D.R., Rouault T.A. (2012). Both Human Ferredoxins 1 and 2 and Ferredoxin Reductase Are Important for Iron-Sulfur Cluster Biogenesis. Biochim. Biophys. Acta.

[217] Hanukoglu I. (1992). Steroidogenic Enzymes: Structure, Function, and Role in Regulation of Steroid Hormone Biosynthesis. J. Steroid. Biochem. Mol. Biol..

[218] Wang J., Zhao R., Ma J., Qin J., Zhang H., Guo J., Chang X., Zhang W. (2025). Biallelic FDXR Mutations Induce Ferroptosis in a Rare Mitochondrial Disease with Ataxia. Free Radic. Biol. Med..

[219] Du J., Zhou Y., Li Y., Xia J., Chen Y., Chen S., Wang X., Sun W., Wang T., Ren X. (2020). Identification of Frataxin as a Regulator of Ferroptosis. Redox Biol..

[220] Schulz T.J., Thierbach R., Voigt A., Drewes G., Mietzner B., Steinberg P., Pfeiffer A.F.H., Ristow M. (2006). Induction of Oxidative Metabolism by Mitochondrial Frataxin Inhibits Cancer Growth: Otto Warburg Revisited. J. Biol. Chem..

[221] Thierbach R., Schulz T.J., Isken F., Voigt A., Mietzner B., Drewes G., von Kleist-Retzow J.-C., Wiesner R.J., Magnuson M.A., Puccio H. (2005). Targeted Disruption of Hepatic Frataxin Expression Causes Impaired Mitochondrial Function, Decreased Life Span and Tumor Growth in Mice. Hum. Mol. Genet..

[222] Guccini I., Serio D., Condò I., Rufini A., Tomassini B., Mangiola A., Maira G., Anile C., Fina D., Pallone F. (2011). Frataxin Participates to the Hypoxia-Induced Response in Tumors. Cell Death Dis..

[223] Amaral N., Okonko D.O. (2015). Mitigation of Myocardial Ischemia-Reperfusion Injury via HIF-1α-Frataxin Signaling. Am. J. Physiol. Heart Circ. Physiol..

[224] Bensaad K., Tsuruta A., Selak M.A., Vidal M.N.C., Nakano K., Bartrons R., Gottlieb E., Vousden K.H. (2006). TIGAR, a P53-Inducible Regulator of Glycolysis and Apoptosis. Cell.

[225] Bensaad K., Cheung E.C., Vousden K.H. (2009). Modulation of Intracellular ROS Levels by TIGAR Controls Autophagy. EMBO J..

[226] Wanka C., Steinbach J.P., Rieger J. (2012). Tp53-Induced Glycolysis and Apoptosis Regulator (TIGAR) Protects Glioma Cells from Starvation-Induced Cell Death by up-Regulating Respiration and Improving Cellular Redox Homeostasis. J. Biol. Chem..

[227] Cheung E.C., Athineos D., Lee P., Ridgway R.A., Lambie W., Nixon C., Strathdee D., Blyth K., Sansom O.J., Vousden K.H. (2013). TIGAR Is Required for Efficient Intestinal Regeneration and Tumorigenesis. Dev. Cell.

[228] Ye L., Zhao X., Lu J., Qian G., Zheng J.C., Ge S. (2013). Knockdown of TIGAR by RNA Interference Induces Apoptosis and Autophagy in HepG2 Hepatocellular Carcinoma Cells. Biochem. Biophys. Res. Commun..

[229] Chen L., Zhang Z., Hoshino A., Zheng H.D., Morley M., Arany Z., Rabinowitz J.D. (2019). NADPH Production by the Oxidative Pentose-Phosphate Pathway Supports Folate Metabolism. Nat. Metab..

[230] Li H., Jogl G. (2009). Structural and Biochemical Studies of TIGAR (TP53-Induced Glycolysis and Apoptosis Regulator). J. Biol. Chem..

[231] Liu M.-Y., Li H.-M., Wang X.-Y., Xia R., Li X., Ma Y.-J., Wang M., Zhang H.-S. (2022). TIGAR Drives Colorectal Cancer Ferroptosis Resistance through ROS/AMPK/SCD1 Pathway. Free Radic. Biol. Med..

[232] Nagy P. (2013). Kinetics and Mechanisms of Thiol-Disulfide Exchange Covering Direct Substitution and Thiol Oxidation-Mediated Pathways. Antioxid. Redox Signal..

[233] Ren X., Zou L., Zhang X., Branco V., Wang J., Carvalho C., Holmgren A., Lu J. (2017). Redox Signaling Mediated by Thioredoxin and Glutathione Systems in the Central Nervous System. Antioxid. Redox Signal..

[234] Forshaw T.E., Holmila R., Nelson K.J., Lewis J.E., Kemp M.L., Tsang A.W., Poole L.B., Lowther W.T., Furdui C.M. (2019). Peroxiredoxins in Cancer and Response to Radiation Therapies. Antioxidants.

[235] Ueno M., Masutani H., Arai R.J., Yamauchi A., Hirota K., Sakai T., Inamoto T., Yamaoka Y., Yodoi J., Nikaido T. (1999). Thioredoxin-Dependent Redox Regulation of P53-Mediated P21 Activation. J. Biol. Chem..

[236] Jayaraman L., Murthy K.G., Zhu C., Curran T., Xanthoudakis S., Prives C. (1997). Identification of Redox/Repair Protein Ref-1 as a Potent Activator of P53. Genes Dev..

[237] Hainaut P., Mann K. (2001). Zinc Binding and Redox Control of P53 Structure and Function. Antioxid. Redox Signal..

[238] Seemann S., Hainaut P. (2005). Roles of Thioredoxin Reductase 1 and APE/Ref-1 in the Control of Basal P53 Stability and Activity. Oncogene.

[239] Velasco-Miguel S., Buckbinder L., Jean P., Gelbert L., Talbott R., Laidlaw J., Seizinger B., Kley N. (1999). PA26, a Novel Target of the P53 Tumor Suppressor and Member of the GADD Family of DNA Damage and Growth Arrest Inducible Genes. Oncogene.

[240] Budanov A.V., Shoshani T., Faerman A., Zelin E., Kamer I., Kalinski H., Gorodin S., Fishman A., Chajut A., Einat P. (2002). Identification of a Novel Stress-Responsive Gene Hi95 Involved in Regulation of Cell Viability. Oncogene.

[241] Budanov A.V. (2011). Stress-Responsive Sestrins Link P53 with Redox Regulation and Mammalian Target of Rapamycin Signaling. Antioxid. Redox Signal..

[242] Budanov A.V., Sablina A.A., Feinstein E., Koonin E.V., Chumakov P.M. (2004). Regeneration of Peroxiredoxins by P53-Regulated Sestrins, Homologs of Bacterial AhpD. Science.

[243] Bae S.H., Sung S.H., Oh S.Y., Lim J.M., Lee S.K., Park Y.N., Lee H.E., Kang D., Rhee S.G. (2013). Sestrins Activate Nrf2 by Promoting P62-Dependent Autophagic Degradation of Keap1 and Prevent Oxidative Liver Damage. Cell Metab..

[244] Zhang L., Ding K., Liao S.-S., Zhang Y.-G., Liao H.-Y., Chen R., Meng Q.-T. (2024). Sestrin2 Reduces Ferroptosis via the Keap1/Nrf2 Signaling Pathway after Intestinal Ischemia-Reperfusion. Free Radic. Biol. Med..

[245] Tomasini R., Samir A.A., Vaccaro M.I., Pebusque M.J., Dagorn J.C., Iovanna J.L., Dusetti N.J. (2001). Molecular and Functional Characterization of the Stress-Induced Protein (SIP) Gene and Its Two Transcripts Generated by Alternative Splicing. SIP Induced by Stress and Promotes Cell Death. J. Biol. Chem..

[246] Okamura S., Arakawa H., Tanaka T., Nakanishi H., Ng C.C., Taya Y., Monden M., Nakamura Y. (2001). p53DINP1, a P53-Inducible Gene, Regulates P53-Dependent Apoptosis. Mol. Cell.

[247] Tomasini R., Samir A.A., Pebusque M.-J., Calvo E.L., Totaro S., Dagorn J.C., Dusetti N.J., Iovanna J.L. (2002). P53-Dependent Expression of the Stress-Induced Protein (SIP). Eur. J. Cell Biol..

[248] Cano C.E., Gommeaux J., Pietri S., Culcasi M., Garcia S., Seux M., Barelier S., Vasseur S., Spoto R.P., Pébusque M.-J. (2009). Tumor Protein 53-Induced Nuclear Protein 1 Is a Major Mediator of P53 Antioxidant Function. Cancer Res..

[249] Tomasini R., Samir A.A., Carrier A., Isnardon D., Cecchinelli B., Soddu S., Malissen B., Dagorn J.-C., Iovanna J.L., Dusetti N.J. (2003). TP53INP1s and Homeodomain-Interacting Protein Kinase-2 (HIPK2) Are Partners in Regulating P53 Activity. J. Biol. Chem..

[250] Yoshida K., Liu H., Miki Y. (2006). Protein Kinase C Delta Regulates Ser46 Phosphorylation of P53 Tumor Suppressor in the Apoptotic Response to DNA Damage. J. Biol. Chem..

[251] Manohar S., Estrada M.E., Uliana F., Vuina K., Alvarez P.M., de Bruin R.A.M., Neurohr G.E. (2023). Genome Homeostasis Defects Drive Enlarged Cells into Senescence. Mol. Cell.

[252] O’Reilly M.A. (2005). Redox Activation of p21Cip1/WAF1/Sdi1: A Multifunctional Regulator of Cell Survival and Death. Antioxid. Redox Signal..

[253] Maddocks O.D.K., Berkers C.R., Mason S.M., Zheng L., Blyth K., Gottlieb E., Vousden K.H. (2013). Serine Starvation Induces Stress and P53-Dependent Metabolic Remodelling in Cancer Cells. Nature.

[254] Wu M., Xu L.-G., Li X., Zhai Z., Shu H.-B. (2002). AMID, an Apoptosis-Inducing Factor-Homologous Mitochondrion-Associated Protein, Induces Caspase-Independent Apoptosis. J. Biol. Chem..

[255] Marshall K.R., Gong M., Wodke L., Lamb J.H., Jones D.J.L., Farmer P.B., Scrutton N.S., Munro A.W. (2005). The Human Apoptosis-Inducing Protein AMID Is an Oxidoreductase with a Modified Flavin Cofactor and DNA Binding Activity. J. Biol. Chem..

[256] Dai E., Meng L., Kang R., Wang X., Tang D. (2020). ESCRT-III-Dependent Membrane Repair Blocks Ferroptosis. Biochem. Biophys. Res. Commun..

[257] Xie Y., Zhu S., Song X., Sun X., Fan Y., Liu J., Zhong M., Yuan H., Zhang L., Billiar T.R. (2017). The Tumor Suppressor P53 Limits Ferroptosis by Blocking DPP4 Activity. Cell. Rep..

[258] Chen D., Chu B., Yang X., Liu Z., Jin Y., Kon N., Rabadan R., Jiang X., Stockwell B.R., Gu W. (2021). iPLA2β-Mediated Lipid Detoxification Controls P53-Driven Ferroptosis Independent of GPX4. Nat. Commun..

[259] Sun W.-Y., Tyurin V.A., Mikulska-Ruminska K., Shrivastava I.H., Anthonymuthu T.S., Zhai Y.-J., Pan M.-H., Gong H.-B., Lu D.-H., Sun J. (2021). Phospholipase iPLA2β Averts Ferroptosis by Eliminating a Redox Lipid Death Signal. Nat. Chem. Biol..

[260] Tornesello M.L. (2025). TP53 Mutations in Cancer: Molecular Features and Therapeutic Opportunities (Review). Int. J. Mol. Med..

[261] Wang D., Nakayama M., Hong C.P., Oshima H., Oshima M. (2024). Gain-of-Function P53 Mutation Acts as a Genetic Switch for TGFβ Signaling-Induced Epithelial-to-Mesenchymal Transition in Intestinal Tumors. Cancer Res..

[262] Alvarado-Ortiz E., de la Cruz-López K.G., Becerril-Rico J., Sarabia-Sánchez M.A., Ortiz-Sánchez E., García-Carrancá A. (2020). Mutant P53 Gain-of-Function: Role in Cancer Development, Progression, and Therapeutic Approaches. Front. Cell. Dev. Biol..

[263] Kastenhuber E.R., Lowe S.W. (2017). Putting P53 in Context. Cell.

[264] Levine A.J., Berger S.L. (2017). The Interplay between Epigenetic Changes and the P53 Protein in Stem Cells. Genes Dev..

[265] Li T., Kon N., Jiang L., Tan M., Ludwig T., Zhao Y., Baer R., Gu W. (2012). Tumor Suppression in the Absence of P53-Mediated Cell-Cycle Arrest, Apoptosis, and Senescence. Cell.

[266] Liu D.S., Duong C.P., Haupt S., Montgomery K.G., House C.M., Azar W.J., Pearson H.B., Fisher O.M., Read M., Guerra G.R. (2017). Inhibiting the System xC-/Glutathione Axis Selectively Targets Cancers with Mutant-P53 Accumulation. Nat. Commun..

[267] Wang S.-J., Li D., Ou Y., Jiang L., Chen Y., Zhao Y., Gu W. (2016). Acetylation Is Crucial for P53-Mediated Ferroptosis and Tumor Suppression. Cell Rep..

[268] Dibra D., Moyer S.M., El-Naggar A.K., Qi Y., Su X., Lozano G. (2023). Triple-Negative Breast Tumors Are Dependent on Mutant P53 for Growth and Survival. Proc. Natl. Acad. Sci. USA.

[269] Dibra D., Xiong S., Moyer S.M., El-Naggar A.K., Qi Y., Su X., Kong E.K., Korkut A., Lozano G. (2024). Mutant P53 Protects Triple-Negative Breast Adenocarcinomas from Ferroptosis In Vivo. Sci. Adv..

[270] Li X.-L., Zhou J., Chan Z.-L., Chooi J.-Y., Chen Z.-R., Chng W.-J. (2015). PRIMA-1met (APR-246) Inhibits Growth of Colorectal Cancer Cells with Different P53 Status through Distinct Mechanisms. Oncotarget.

[271] Sallman D.A., DeZern A.E., Garcia-Manero G., Steensma D.P., Roboz G.J., Sekeres M.A., Cluzeau T., Sweet K.L., McLemore A., McGraw K.L. (2021). Eprenetapopt (APR-246) and Azacitidine in TP53-Mutant Myelodysplastic Syndromes. J. Clin. Oncol..

[272] Cluzeau T., Sebert M., Rahmé R., Cuzzubbo S., Lehmann-Che J., Madelaine I., Peterlin P., Bève B., Attalah H., Chermat F. (2021). Eprenetapopt Plus Azacitidine in TP53-Mutated Myelodysplastic Syndromes and Acute Myeloid Leukemia: A Phase II Study by the Groupe Francophone Des Myélodysplasies (GFM). J. Clin. Oncol..

[273] Birsen R., Larrue C., Decroocq J., Johnson N., Guiraud N., Gotanegre M., Cantero-Aguilar L., Grignano E., Huynh T., Fontenay M. (2022). APR-246 Induces Early Cell Death by Ferroptosis in Acute Myeloid Leukemia. Haematologica.

[274] Lei G., Zhang Y., Hong T., Zhang X., Liu X., Mao C., Yan Y., Koppula P., Cheng W., Sood A.K. (2021). Ferroptosis as a Mechanism to Mediate P53 Function in Tumor Radiosensitivity. Oncogene.

[275] Fan X., Ou Y., Liu H., Zhan L., Zhu X., Cheng M., Li Q., Yin D., Liao L. (2021). A Ferroptosis-Related Prognostic Signature Based on Antitumor Immunity and Tumor Protein P53 Mutation Exploration for Guiding Treatment in Patients With Head and Neck Squamous Cell Carcinoma. Front. Genet..

[276] Ru Q., Li Y., Chen L., Wu Y., Min J., Wang F. (2024). Iron Homeostasis and Ferroptosis in Human Diseases: Mechanisms and Therapeutic Prospects. Signal Transduct. Target. Ther..

[277] Bao W.-D., Zhou X.-T., Zhou L.-T., Wang F., Yin X., Lu Y., Zhu L.-Q., Liu D. (2020). Targeting miR-124/Ferroportin Signaling Ameliorated Neuronal Cell Death through Inhibiting Apoptosis and Ferroptosis in Aged Intracerebral Hemorrhage Murine Model. Aging Cell.

[278] Babu K.R., Muckenthaler M.U. (2016). miR-20a Regulates Expression of the Iron Exporter Ferroportin in Lung Cancer. J. Mol. Med..

[279] Kong Y., Hu L., Lu K., Wang Y., Xie Y., Gao L., Yang G., Xie B., He W., Chen G. (2019). Ferroportin Downregulation Promotes Cell Proliferation by Modulating the Nrf2–miR-17-5p Axis in Multiple Myeloma. Cell Death Dis..

[280] Xu P., Ge F.-H., Li W.-X., Xu Z., Wang X.-L., Shen J.-L., Xu A.-B., Hao R.-R. (2022). MicroRNA-147a Targets SLC40A1 to Induce Ferroptosis in Human Glioblastoma. Anal. Cell. Pathol..

[281] Zhu C., Song Z., Chen Z., Lin T., Lin H., Xu Z., Ai F., Zheng S. (2022). MicroRNA-4735-3p Facilitates Ferroptosis in Clear Cell Renal Cell Carcinoma by Targeting SLC40A1. Anal. Cell. Pathol..

[282] Wei D., Ke Y.-Q., Duan P., Zhou L., Wang C.-Y., Cao P. (2021). MicroRNA-302a-3p Induces Ferroptosis of Non-Small Cell Lung Cancer Cells via Targeting Ferroportin. Free Radic. Res..

[283] Jiang Z., Zhou J., Deng J., Li L., Wang R., Han Y., Zhou J., Tao R., Peng L., Wang D. (2023). Emerging Roles of Ferroptosis-Related miRNAs in Tumor Metastasis. Cell Death Discov..

[284] Zheng X., Zhang C. (2023). The Regulation of Ferroptosis by Noncoding RNAs. Int. J. Mol. Sci..

[285] Zheng R., Lin C., Mao Y., Jin F. (2023). miR-761-Hepcidin/Gpx4 Pathway Contribute to Unexplained Liver Dysfunction in Polycystic Ovary Syndrome by Regulating Liver Iron Overload and Ferroptosis. Gynecol. Endocrinol..

[286] Tomita K., Fukumoto M., Itoh K., Kuwahara Y., Igarashi K., Nagasawa T., Suzuki M., Kurimasa A., Sato T. (2019). MiR-7-5p Is a Key Factor That Controls Radioresistance via Intracellular Fe2+ Content in Clinically Relevant Radioresistant Cells. Biochem. Biophys. Res. Commun..

[287] Li X., Si W., Li Z., Tian Y., Liu X., Ye S., Huang Z., Ji Y., Zhao C., Hao X. (2021). miR-335 Promotes Ferroptosis by Targeting Ferritin Heavy Chain 1 in in Vivo and in Vitro Models of Parkinson’s Disease. Int. J. Mol. Med..

[288] Zheng H., Shi L., Tong C., Liu Y., Hou M. (2021). circSnx12 Is Involved in Ferroptosis During Heart Failure by Targeting miR-224-5p. Front. Cardiovasc. Med..

[289] Zhang R., Pan T., Xiang Y., Zhang M., Xie H., Liang Z., Chen B., Xu C., Wang J., Huang X. (2022). Curcumenol Triggered Ferroptosis in Lung Cancer Cells via lncRNA H19/miR-19b-3p/FTH1 Axis. Bioact. Mater..

[290] Lei D., Li B., Isa Z., Ma X., Zhang B. (2022). Hypoxia-Elicited Cardiac Microvascular Endothelial Cell-Derived Exosomal miR-210-3p Alleviate Hypoxia/Reoxygenation-Induced Myocardial Cell Injury through Inhibiting Transferrin Receptor 1-Mediated Ferroptosis. Tissue Cell.

[291] Zhao D., Ji H., Zhang W., He A., Guo C., Ma L., Liu Y. (2025). miR-214-3p Inhibits LPS-Induced Macrophage Inflammation and Attenuates the Progression of Dry Eye Syndrome by Regulating Ferroptosis in Cells. Genes Genomics.

[292] Huang J., Deng C., Guo T., Chen X., Chen P., Du S., Lu M. (2023). Cinobufotalin Induces Ferroptosis to Suppress Lung Cancer Cell Growth by lncRNA LINC00597/Hsa-miR-367-3p/TFRC Pathway via Resibufogenin. Anticancer Agents Med. Chem..

[293] Ajam-Hosseini M., Babashah S. (2025). Exploring Ferroptosis and miRNAs: Implications for Cancer Modulation and Therapy. Mol. Cell. Biochem..

[294] Scarpellini C., Klejborowska G., Lanthier C., Hassannia B., Vanden Berghe T., Augustyns K. (2023). Beyond Ferrostatin-1: A Comprehensive Review of Ferroptosis Inhibitors. Trends Pharmacol. Sci..

[295] Lu J., Xu F., Lu H. (2020). LncRNA PVT1 Regulates Ferroptosis through miR-214-Mediated TFR1 and P53. Life Sci..

[296] Chaudhary R., Lal A. (2017). Long Noncoding RNAs in the P53 Network. Wiley Interdiscip. Rev. RNA.

[297] Chen H., Han Z., Su J., Song X., Ma Q., Lin Y., Ran Z., Li X., Mou R., Wang Y. (2024). Ferroptosis and Hepatocellular Carcinoma: The Emerging Role of lncRNAs. Front. Immunol..

[298] Zhang J., Chen S., Wei S., Cheng S., Shi R., Zhao R., Zhang W., Zhang Q., Hua T., Feng D. (2022). CircRAPGEF5 Interacts with RBFOX2 to Confer Ferroptosis Resistance by Modulating Alternative Splicing of TFRC in Endometrial Cancer. Redox Biol..

[299] Liu M., Kong X.-Y., Yao Y., Wang X.-A., Yang W., Wu H., Li S., Ding J.-W., Yang J. (2022). The Critical Role and Molecular Mechanisms of Ferroptosis in Antioxidant Systems: A Narrative Review. Ann. Transl. Med..

[300] Zhou Q., Meng Y., Li D., Yao L., Le J., Liu Y., Sun Y., Zeng F., Chen X., Deng G. (2024). Ferroptosis in Cancer: From Molecular Mechanisms to Therapeutic Strategies. Signal Transduct. Target. Ther..

[301] Riccio P., Sessa R., de Nicola S., Petruzziello F., Trombetti S., Menna G., Pepe G., Maddalena P., Izzo P., Grosso M. (2019). GATA-1 Isoforms Differently Contribute to the Production and Compartmentation of Reactive Oxygen Species in the Myeloid Leukemia Cell Line K562. J. Cell. Physiol..

[302] Trombetti S., Sessa R., Catapano R., Rinaldi L., Lo Bianco A., Feliciello A., Izzo P., Grosso M. (2021). Exploring the Leukemogenic Potential of GATA-1S, the Shorter Isoform of GATA-1: Novel Insights into Mechanisms Hampering Respiratory Chain Complex II Activity and Limiting Oxidative Phosphorylation Efficiency. Antioxidants.

[303] Trombetti S., Iaccarino N., Riccio P., Sessa R., Catapano R., Salvatore M., Luka S., de Nicola S., Izzo P., Roperto S. (2023). Over-Expressed GATA-1S, the Short Isoform of the Hematopoietic Transcriptional Factor GATA-1, Inhibits Ferroptosis in K562 Myeloid Leukemia Cells by Preventing Lipid Peroxidation. Antioxidants.

[304] Montano G., Vidovic K., Palladino C., Cesaro E., Sodaro G., Quintarelli C., De Angelis B., Errichiello S., Pane F., Izzo P. (2015). WT1-Mediated Repression of the Proapoptotic Transcription Factor ZNF224 Is Triggered by the BCR-ABL Oncogene. Oncotarget.

[305] Balihodzic A., Prinz F., Dengler M.A., Calin G.A., Jost P.J., Pichler M. (2022). Non-Coding RNAs and Ferroptosis: Potential Implications for Cancer Therapy. Cell Death Differ..

[306] Piccolo M., Ferraro M.G., Iazzetti F., Santamaria R., Irace C. (2024). Insight into Iron, Oxidative Stress and Ferroptosis: Therapy Targets for Approaching Anticancer Strategies. Cancers.

[307] Jiang Y., Saeed T.N., Alfarttoosi K.H., Bishoyi A.K., Rekha M.M., Kundlas M., Jain B., Rizaev J., Taher W.M., Alwan M. (2025). The Intersection of Ferroptosis and Non-Coding RNAs: A Novel Approach to Ovarian Cancer. Eur. J. Med. Res..

[308] Zhang L., Hou N., Chen B., Kan C., Han F., Zhang J., Sun X. (2022). Post-Translational Modifications of P53 in Ferroptosis: Novel Pharmacological Targets for Cancer Therapy. Front. Pharmacol..

[309] Shang Z., Luo Z., Wang Y., Liu Q., Xin Y., Zhang M., Li X., Zeng S., Yu L., Zhang X. (2023). CircHIPK3 Contributes to Cisplatin Resistance in Gastric Cancer by Blocking Autophagy-Dependent Ferroptosis. J. Cell. Physiol..

[310] Qin K., Zhang F., Wang H., Wang N., Qiu H., Jia X., Gong S., Zhang Z. (2023). circRNA circSnx12 Confers Cisplatin Chemoresistance to Ovarian Cancer by Inhibiting Ferroptosis through a miR-194-5p/SLC7A11 Axis. BMB Rep..

[311] Ma Y., Gao J., Guo H. (2023). Circ_0000140 Alters miR-527/SLC7A11-Mediated Ferroptosis to Influence Oral Squamous Cell Carcinoma Cell Resistance to DDP. Pharmgenomics Pers. Med..

[312] Dong F.-L., Xu Z.-Z., Wang Y.-Q., Li T., Wang X., Li J. (2024). Exosome-Derived circUPF2 Enhances Resistance to Targeted Therapy by Redeploying Ferroptosis Sensitivity in Hepatocellular Carcinoma. J. Nanobiotechnol..

[313] Chen S., Zhang Z., Zhang B., Huang Q., Liu Y., Qiu Y., Long X., Wu M., Zhang Z. (2022). CircCDK14 Promotes Tumor Progression and Resists Ferroptosis in Glioma by Regulating PDGFRA. Int. J. Biol. Sci..

[314] Yao W., Wang J., Meng F., Zhu Z., Jia X., Xu L., Zhang Q., Wei L. (2021). Circular RNA CircPVT1 Inhibits 5-Fluorouracil Chemosensitivity by Regulating Ferroptosis Through MiR-30a-5p/FZD3 Axis in Esophageal Cancer Cells. Front. Oncol..

[315] Wang S., Wang Y., Li Q., Li X., Feng X. (2022). A Novel Circular RNA Confers Trastuzumab Resistance in Human Epidermal Growth Factor Receptor 2-Positive Breast Cancer through Regulating Ferroptosis. Environ. Toxicol..

[316] Lyu N., Zeng Y., Kong Y., Chen Q., Deng H., Ou S., Bai Y., Tang H., Wang X., Zhao M. (2021). Ferroptosis Is Involved in the Progression of Hepatocellular Carcinoma through the Circ0097009/miR-1261/SLC7A11 Axis. Ann. Transl. Med..

[317] Wu P., Li C., Ye D.M., Yu K., Li Y., Tang H., Xu G., Yi S., Zhang Z. (2021). Circular RNA circEPSTI1 Accelerates Cervical Cancer Progression via miR-375/409-3P/515-5p-SLC7A11 Axis. Aging.

[318] Mao C., Wang X., Liu Y., Wang M., Yan B., Jiang Y., Shi Y., Shen Y., Liu X., Lai W. (2018). A G3BP1-Interacting lncRNA Promotes Ferroptosis and Apoptosis in Cancer via Nuclear Sequestration of P53. Cancer Res..

[319] Wang Z., Chen X., Liu N., Shi Y., Liu Y., Ouyang L., Tam S., Xiao D., Liu S., Wen F. (2021). A Nuclear Long Non-Coding RNA LINC00618 Accelerates Ferroptosis in a Manner Dependent upon Apoptosis. Mol. Ther..

[320] Zhang B., Bao W., Zhang S., Chen B., Zhou X., Zhao J., Shi Z., Zhang T., Chen Z., Wang L. (2022). LncRNA HEPFAL Accelerates Ferroptosis in Hepatocellular Carcinoma by Regulating SLC7A11 Ubiquitination. Cell Death Dis..

[321] Jiang X., Guo S., Xu M., Ma B., Liu R., Xu Y., Zhang Y. (2022). TFAP2C-Mediated lncRNA PCAT1 Inhibits Ferroptosis in Docetaxel-Resistant Prostate Cancer Through c-Myc/miR-25-3p/SLC7A11 Signaling. Front. Oncol..

[322] Li L., Zhang Y., Gao Y., Hu Y., Wang R., Wang S., Li Y., He Y., Yuan C. (2023). LncSNHG14 Promotes Nutlin3a Resistance by Inhibiting Ferroptosis via the miR-206 /SLC7A11 Axis in Osteosarcoma Cells. Cancer Gene Ther..

[323] Zong K., Lin C., Luo K., Deng Y., Wang H., Hu J., Chen S., Li R. (2024). Ferroptosis-Related lncRNA NRAV Affects the Prognosis of Hepatocellular Carcinoma via the miR-375-3P/SLC7A11 Axis. BMC Cancer.

[324] Shi Z., Li Z., Jin B., Ye W., Wang L., Zhang S., Zheng J., Lin Z., Chen B., Liu F. (2023). Loss of LncRNA DUXAP8 Synergistically Enhanced Sorafenib Induced Ferroptosis in Hepatocellular Carcinoma via SLC7A11 De-Palmitoylation. Clin. Transl. Med..

[325] Zhang Y., Guo S., Wang S., Li X., Hou D., Li H., Wang L., Xu Y., Ma B., Wang H. (2021). LncRNA OIP5-AS1 Inhibits Ferroptosis in Prostate Cancer with Long-Term Cadmium Exposure through miR-128-3p/SLC7A11 Signaling. Ecotoxicol. Environ. Saf..

[326] Ni H., Qin H., Sun C., Liu Y., Ruan G., Guo Q., Xi T., Xing Y., Zheng L. (2021). MiR-375 Reduces the Stemness of Gastric Cancer Cells through Triggering Ferroptosis. Stem Cell Res. Ther..

[327] Yadav P., Sharma P., Sundaram S., Venkatraman G., Bera A.K., Karunagaran D. (2021). SLC7A11/ xCT Is a Target of miR-5096 and Its Restoration Partially Rescues miR-5096-Mediated Ferroptosis and Anti-Tumor Effects in Human Breast Cancer Cells. Cancer Lett..

[328] Sun K., Ren W., Li S., Zheng J., Huang Y., Zhi K., Gao L. (2022). MiR-34c-3p Upregulates Erastin-Induced Ferroptosis to Inhibit Proliferation in Oral Squamous Cell Carcinomas by Targeting SLC7A11. Pathol. Res. Pract..

[329] Liu X., Huang Z., Chen Q., Chen K., Liu W., Liu G., Chu X., Li D., Ma Y., Tian X. (2024). Hypoxia-Induced Epigenetic Regulation of miR-485-3p Promotes Stemness and Chemoresistance in Pancreatic Ductal Adenocarcinoma via SLC7A11-Mediated Ferroptosis. Cell Death Discov..

[330] Lu X., Kang N., Ling X., Pan M., Du W., Gao S. (2021). MiR-27a-3p Promotes Non-Small Cell Lung Cancer Through SLC7A11-Mediated-Ferroptosis. Front. Oncol..

[331] Shanshan W., Hongying M., Jingjing F., Yiming Y., Yu R., Rui Y. (2021). CircDTL Functions as an Oncogene and Regulates Both Apoptosis and Ferroptosis in Non-Small Cell Lung Cancer Cells. Front. Genet..

[332] Wu N., Zhu D., Li J., Li X., Zhu Z., Rao Q., Hu B., Wang H., Zhu Y. (2023). CircOMA1 Modulates Cabergoline Resistance by Downregulating Ferroptosis in Prolactinoma. J. Endocrinol. Investig..

[333] Chen W., Fu J., Chen Y., Li Y., Ning L., Huang D., Yan S., Zhang Q. (2021). Circular RNA circKIF4A Facilitates the Malignant Progression and Suppresses Ferroptosis by Sponging miR-1231 and Upregulating GPX4 in Papillary Thyroid Cancer. Aging.

[334] Zhu J., Yu Z., Wang X., Zhang J., Chen Y., Chen K., Zhang B., Sun J., Jiang J., Zheng S. (2024). LncRNA MACC1-AS1 Induces Gemcitabine Resistance in Pancreatic Cancer Cells through Suppressing Ferroptosis. Cell Death Discov..

[335] Li X., Li Y., Lian P., Lv Q., Liu F. (2023). Silencing lncRNA HCG18 Regulates GPX4-Inhibited Ferroptosis by Adsorbing miR-450b-5p to Avert Sorafenib Resistance in Hepatocellular Carcinoma. Hum. Exp. Toxicol..

[336] He G.-N., Bao N.-R., Wang S., Xi M., Zhang T.-H., Chen F.-S. (2021). Ketamine Induces Ferroptosis of Liver Cancer Cells by Targeting lncRNA PVT1/miR-214-3p/GPX4. Drug Des. Dev. Ther..

[337] Gomaa A., Peng D., Chen Z., Soutto M., Abouelezz K., Corvalan A., El-Rifai W. (2019). Epigenetic Regulation of AURKA by miR-4715-3p in Upper Gastrointestinal Cancers. Sci. Rep..

[338] Deng S.-H., Wu D.-M., Li L., Liu T., Zhang T., Li J., Yu Y., He M., Zhao Y.-Y., Han R. (2021). miR-324-3p Reverses Cisplatin Resistance by Inducing GPX4-Mediated Ferroptosis in Lung Adenocarcinoma Cell Line A549. Biochem. Biophys. Res. Commun..

[339] Hou Y., Cai S., Yu S., Lin H. (2021). Metformin Induces Ferroptosis by Targeting miR-324-3p/GPX4 Axis in Breast Cancer. Acta Biochim. Biophys. Sin..

[340] Xu Z., Chen L., Wang C., Zhang L., Xu W. (2021). MicroRNA-1287-5p Promotes Ferroptosis of Osteosarcoma Cells through Inhibiting GPX4. Free Radic. Res..

[341] Xu P., Wang Y., Deng Z., Tan Z., Pei X. (2022). MicroRNA-15a Promotes Prostate Cancer Cell Ferroptosis by Inhibiting GPX4 Expression. Oncol. Lett..

[342] Liu L., Yao H., Zhou X., Chen J., Chen G., Shi X., Wu G., Zhou G., He S. (2022). MiR-15a-3p Regulates Ferroptosis via Targeting Glutathione Peroxidase GPX4 in Colorectal Cancer. Mol. Carcinog..

[343] Han B., Liu Y., Zhang Q., Liang L. (2023). Propofol Decreases Cisplatin Resistance of Non-Small Cell Lung Cancer by Inducing GPX4-Mediated Ferroptosis through the miR-744-5p/miR-615-3p Axis. J. Proteom..

[344] Lee J., Shin D., Roh J.-L. (2023). Lipid Metabolism Alterations and Ferroptosis in Cancer: Paving the Way for Solving Cancer Resistance. Eur. J. Pharmacol..

[345] Feng H., Stockwell B.R. (2018). Unsolved Mysteries: How Does Lipid Peroxidation Cause Ferroptosis?. PLoS. Biol..

[346] Tomita K., Nagasawa T., Kuwahara Y., Torii S., Igarashi K., Roudkenar M.H., Roushandeh A.M., Kurimasa A., Sato T. (2021). MiR-7-5p Is Involved in Ferroptosis Signaling and Radioresistance Thru the Generation of ROS in Radioresistant HeLa and SAS Cell Lines. Int. J. Mol. Sci..

[347] Zhang H., Deng T., Liu R., Ning T., Yang H., Liu D., Zhang Q., Lin D., Ge S., Bai M. (2020). CAF Secreted miR-522 Suppresses Ferroptosis and Promotes Acquired Chemo-Resistance in Gastric Cancer. Mol. Cancer.

[348] Kung Y.-A., Chiang H.-J., Li M.-L., Gong Y.-N., Chiu H.-P., Hung C.-T., Huang P.-N., Huang S.-Y., Wang P.-Y., Hsu T.-A. (2022). Acyl-Coenzyme A Synthetase Long-Chain Family Member 4 Is Involved in Viral Replication Organelle Formation and Facilitates Virus Replication via Ferroptosis. mBio.

[349] Gan B. (2022). ACSL4, PUFA, and Ferroptosis: New Arsenal in Anti-Tumor Immunity. Signal Transduct. Target. Ther..

[350] Hou J., Jiang C., Wen X., Li C., Xiong S., Yue T., Long P., Shi J., Zhang Z. (2022). ACSL4 as a Potential Target and Biomarker for Anticancer: From Molecular Mechanisms to Clinical Therapeutics. Front. Pharmacol..

[351] Otmani K., Lewalle P. (2021). Tumor Suppressor miRNA in Cancer Cells and the Tumor Microenvironment: Mechanism of Deregulation and Clinical Implications. Front. Oncol..

[352] Gambari R., Brognara E., Spandidos D.A., Fabbri E. (2016). Targeting oncomiRNAs and Mimicking Tumor Suppressor miRNAs: Νew Trends in the Development of miRNA Therapeutic Strategies in Oncology (Review). Int. J. Oncol..

[353] Bravo-Vázquez L.A., Méndez-García A., Rodríguez A.L., Sahare P., Pathak S., Banerjee A., Duttaroy A.K., Paul S. (2023). Applications of Nanotechnologies for miRNA-Based Cancer Therapeutics: Current Advances and Future Perspectives. Front. Bioeng. Biotechnol..

